# Articulated rods – a novel class of molecular rods based on oligospiroketals (OSK)

**DOI:** 10.3762/bjoc.11.11

**Published:** 2015-01-16

**Authors:** Pablo Wessig, Roswitha Merkel, Peter Müller

**Affiliations:** 1 Institut für Chemie, Universität Potsdam, Karl-Liebknecht-Str. 24–25, D-14476 Potsdam, Germany; 2 Institut für Biologie/Molekulare Biophysik, Humboldt Universität zu Berlin, Invalidenstr. 42, D-10115 Berlin, Germany

**Keywords:** articulated rods, click chemistry, molecular rods, oligospiroketals, pyrene excimer

## Abstract

We developed a new type of molecular rods consisting of two (or more) rigid units linked by a flexible joint. Consequently we called these constructs articulated rods (ARs). The syntheses of ARs were carried out by a flexible and modular approach providing access to a number of compounds with various functionalizations in terminal positions. First applications were presented with pyrene, cinnamoyl and anthracenyl labelled ARs.

## Introduction

One of the basic principles in living nature is based on shape-persistent and relatively rigid molecular and supramolecular systems. These systems mainly consist of peptides and proteins (e.g., scleroproteins, globular proteins, and membrane proteins), lipids (e.g., biological membranes), carbohydrates (e.g., cellulose) and nucleic acids (DNA, RNA). The impressive performance of the natural systems is achieved by an enormous effort, as measured by the molecular weight of the biomolecules. Mimicking natural construction principles with artificial systems is one of the most challenging tasks of synthetic organic chemistry. In general, the components of these systems are similar to mechanical parts: rods, joints, sleeves, plates etc., but with nanoscopic scale. Amongst these building blocks, molecular rods attracted particular attention in the last past decades [1–6]. Molecular rods, i.e., conformational rigid molecules in which the length considerably exceeds width and height (large aspect ratio), can roughly be divided into conjugated and non-conjugated rods. Whereas the former are of particular interest for nanoelectronics [7], the latter are regularly used in biological applications. Several years ago we developed a new class of molecular rods whose structure is based on spirocyclically joined saturated six-membered rings [8]. The key step of the synthesis of these rods is the formation of ketals and we therefore name this class of compounds oligospiroketals (OSKs).

**Figure 1:**
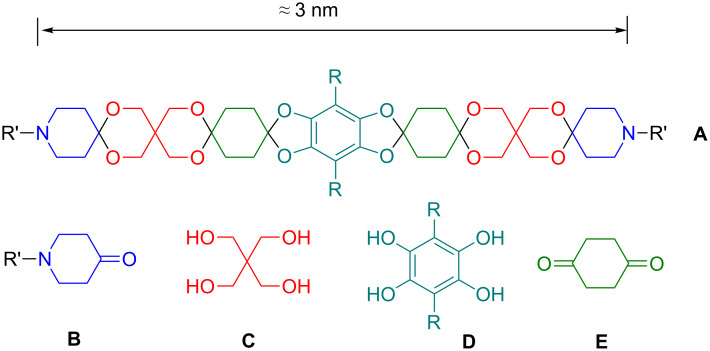
Typical OSK rod **A** with solubility enhancing sleeve (**D**) and building blocks **B**,**C**,**E**.

Although the backbone of OSK rods contains many oxygen atoms, these atoms are shielded by adjacent methylen groups and, consequently, OSK rods are rather hydrophobic. Combined with their rigidity this causes dramatically reduced solubility of longer OSK rods (with more than 6–7 rings). This problem could be circumvented by the introduction of solubility enhancing groups (SEGs), either in terminal or lateral positions of the rods. Building blocks with lateral SEGs are called sleeves. In Figure 1 a typical sleeve **D** (cyan) as well as other typical building blocks of OSK rods are depicted, such as pentaerythritol **C** (red), cyclohexane-1,4-dione **E** (green), and piperidine-4-ones **B** (blue). An OSK rod **A** composed of these structural elements exhibits a length of ca. 3 nm [9]. Later, other sleeves were developed and a method for quantifying the rigidity of molecular rods was established [10].

Several applications of OSK rods were explored in the past years. Worthy of note are the incorporation in biological and artificial membranes [11–13], the utilization as spacer in FRET systems [14], and as building blocks in porous materials [15].

**Figure 2:**
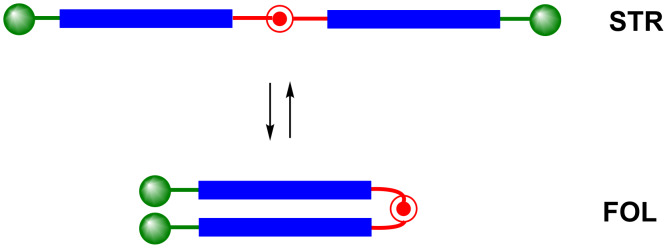
Fundamental structure of articulated rods (blue = legs, red = joint, green = terminal functionalities).

Although stiffness of the whole rod is desirable in many cases, a partial flexibility is often necessary. Especially for biological and biochemical applications this facilitates perfect adjustment between artificial and biological systems. This can be achieved by constructs consisting of rigid legs connected by a flexible joint. An outstanding feature of these systems is the equilibrium between a straight (STR) and a folded (FOL) conformation (Figure 2). Herein we wish to report on the synthesis and properties of a new class of semi-flexible molecules which we called articulated rods (ARs).

## Results and Discussion

### Synthesis of articulated rods (ARs)

The synthetic strategy towards ARs has to address the following issues: i) synthesis of legs (short rods) with orthogonal protecting groups at both ends, ii) selective deprotection/activation of each of these groups, iii) construction of the joint, iv) functionalizing the terminal positions of the ARs, and v) (possibly) introduction of solubility enhancing groups.

**Figure 3:**
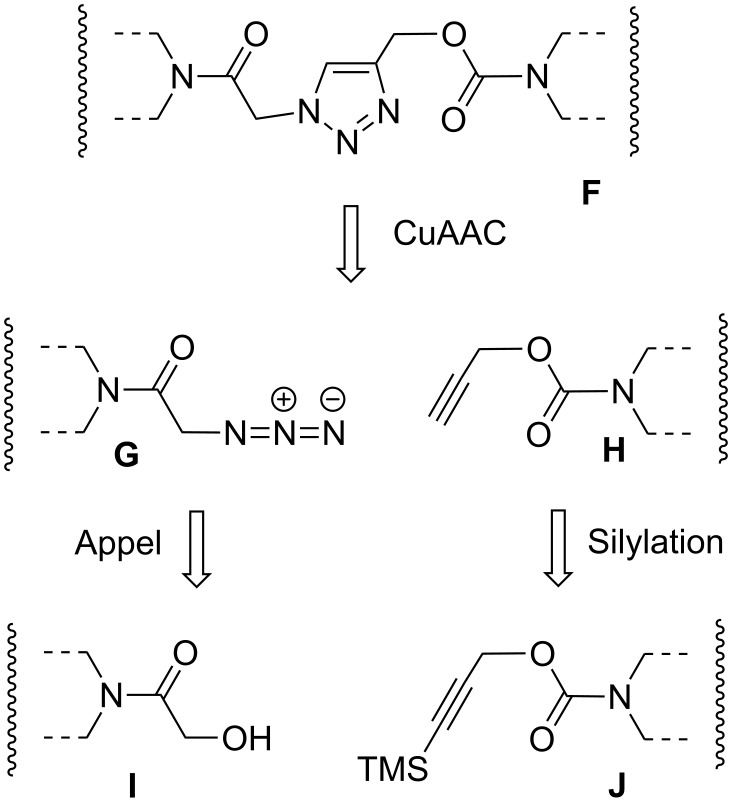
Synthetic strategy towards articulated rods.

Our strategy is summarized in Figure 3. We defined a 1,2,3-triazole containing linkage between two piperidine rings as the flexible joint **F**, which should be easily accessible by copper-catalyzed cycloaddition between an azide **G** and a terminal alkyne **H** (CuAAC, “Click” reaction) [16]. Primary alcohols **I** serve as “protected” azides accessible by modified Appel reaction [17]. The alkynes **H** can be protected by silylation (**J**) because only primary alkynes react in the CuAAC (Figure 3).

**Scheme 1:**
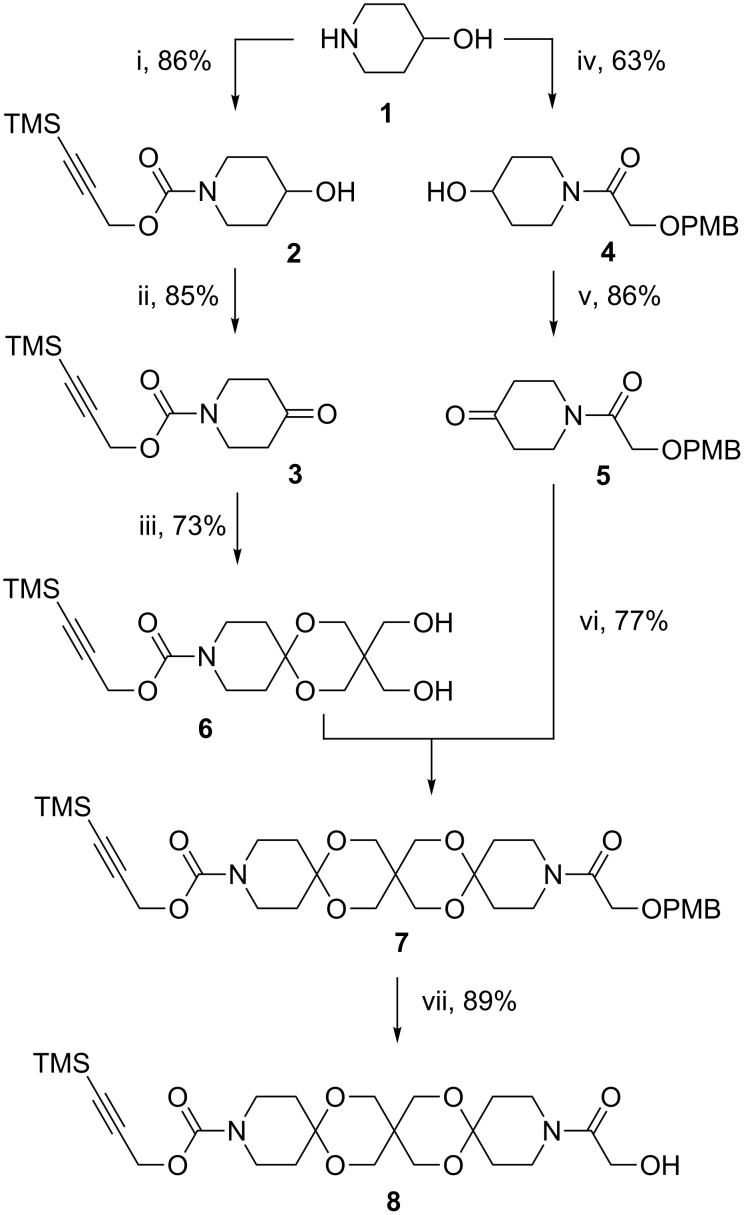
Synthesis of building block **8** (i: trimethylsilylpropargyl-4-nitrophenylcarbonate. ii: Dess–Martin-periodinane. iii: pentaerythritol, pTsOH (cat.). iv: 2-(4-methoxybenzyloxy)acetic acid, DCC, HOBt. v: (COCl)_2_/DMSO. vi: NaH, TMSCl, TMSOTf, vii DDQ, DCM, buffer pH 7).

The synthesis commences with 4-hydroxypiperidine (**1**), which was converted to piperidine-4-one **3** bearing a protected alkyne moiety as well as to piperidin-4-one **5** with a PMB protected hydroxy group, each in two steps. The trispirane **7** was prepared in two steps from ketones **3** and **5** by an acetalization with pentaerythritol under classic conditions followed by reaction of the resulting diol **6** with **5** using our previously developed method of double activation [8]. Finally, the PMB group was cleaved by DDQ to give the orthogonally protected trispiro rod **8** (Scheme 1).

**Scheme 2:**
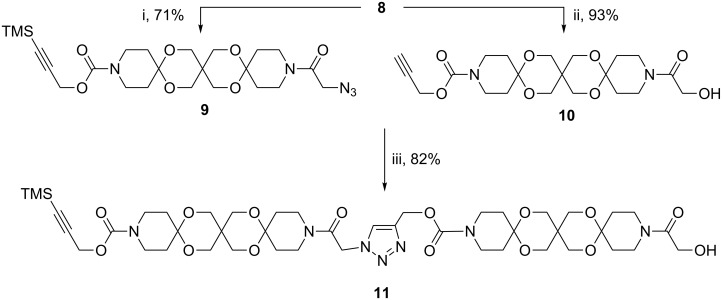
Synthesis of articulated rod **11** (i: CBr_4_, PPh_3_, NaN_3_. ii: K_2_CO_3_/MeOH. iii: Cu/C DCM/MeOH 1:1, cat. Et_3_N).

To demonstrate the capability of the approach outlined in Figure 3 we selectively activated both sides of building block **8** either by introduction of an azide group (**9**) or liberation of the terminal alkyne by desilylation (**10**). The subsequent CuAAC coupling between **9** and **10** turned out to be difficult. Whereas the reaction failed under standard conditions [18] we succeeded after numerous variations of the reaction conditions by applying the Cu/C catalyst [19] in a solvent mixture of DCM and MeOH in the presence of Et_3_N (Scheme 2).

With the first articulated rod **11** in hands we investigated whether it is possible to selectively activate the “click-functionalities” again.

**Scheme 3:**
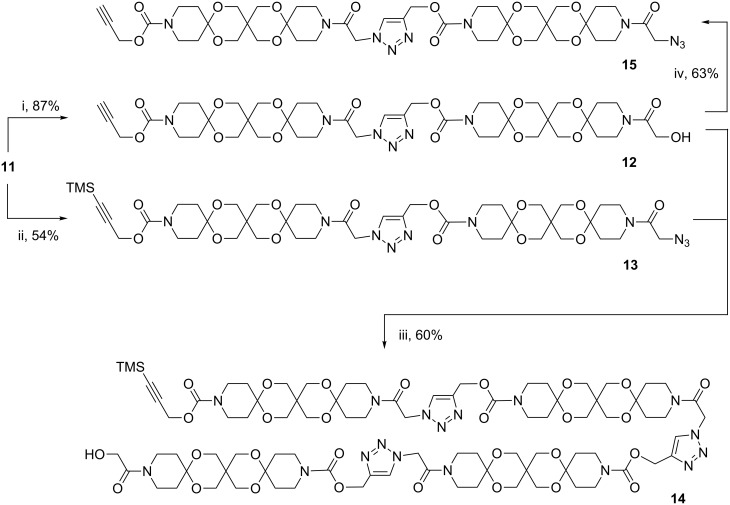
Sequential deprotection of **11** and synthesis of triple articulated rod **14** (i: K_2_CO_3_/MeOH. ii: CBr_4_/PPh_3_/NaN_3_.iii: Cu/C, Et_3_N. iv: CBr_4_/PPh_3_/NaN_3_.).

To our delight **11** could be converted in good yields both to the unprotected alkyne **12** and to the azide **13**. Coupling of these articulated rods using the previously optimized CuAAC conditions afforded the triple articulated rod **14** in satisfactory yields (Scheme 3). It should be noted that the molecule **14** already has a length of nearly 7 nm in the stretched conformation. Furthermore, we activated both ends of **11** in two steps to give the AR **15** and undertook several attempts to cyclize this compound using the hitherto successful catalyst Cu/C. The cyclization of articulated rods could be a promising approach to produce bundles of rods with increased stiffness. Unfortunately, we obtained only insoluble solids, which could not be further characterized. Only with CuBr in the presence of DBU [20] we received evidence for a cyclization of **15** from IR and MS spectra.

At this point we could not rule out that polymerization of **15** occurred instead of the desired cyclization. On the other hand it was obvious that the cyclization product can be insoluble due to the dramatically reduced conformational flexibility.

**Scheme 4:**
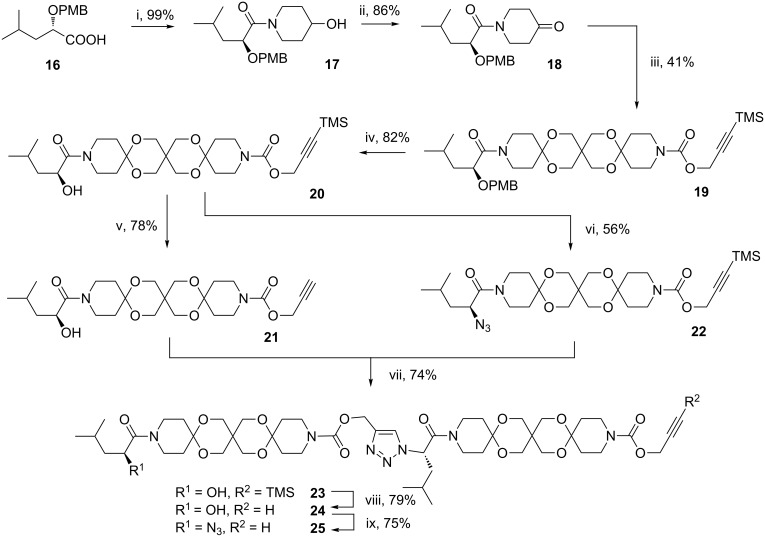
Synthesis of articulated rods **23**–**25** with increased solubility (i: 4-hydroxypiperidine, DCC, HOBt. ii: (COCl)_2_/DMSO. iii: **6**, NaH, TMSCl, TMSOTf. iv: DDQ. v: K_2_CO_3_/MeOH. vi: CBr_4_/PPh_3_. vii: Cu/C, Et_3_N. viii: K_2_CO_3_/MeOH. ix: CBr_4_/PPh_3_/NaN_3_).

**Scheme 5:**
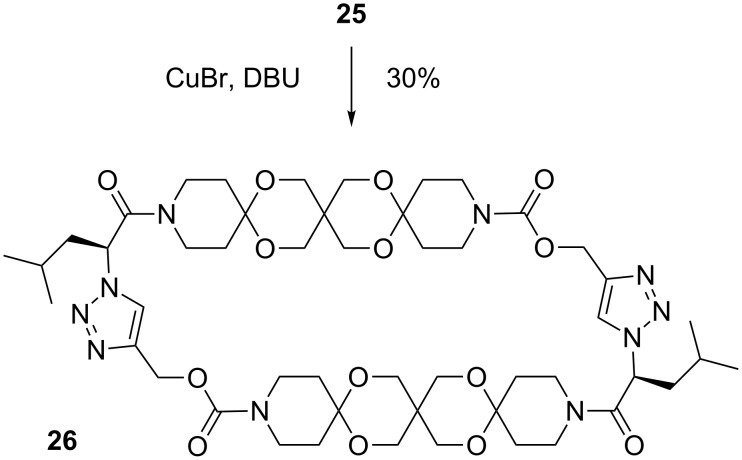
Macrocyclization of articulated rod **25**.

To prove this hypothesis we developed a articulated rod bearing solubility enhancing groups. Starting with PMB-protected and enantiomerically pure 2-hydroxy-4-methylpentanoic acid **16**, which is easily accessible from L-leucine [21] we pursued a similar strategy as described above for articulated rod **15**. Accordingly, articulated rod **25** was obtained in nine steps (Scheme 4). To our delight, **25** could be successfully cyclized to **26** using the above mentioned CuBr/DBU catalyst albeit with moderate yield (Scheme 5).

**Scheme 6:**
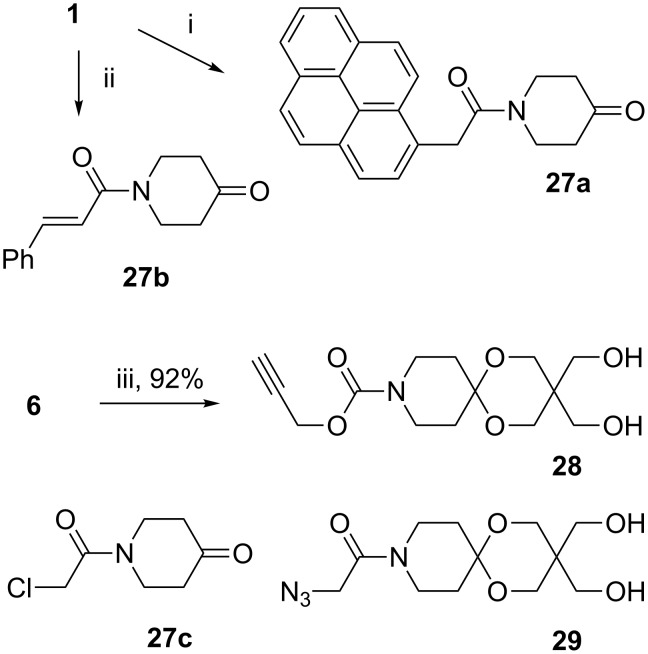
Synthesis of building blocks **27**–**29** (i: 1. pyrene-1-ylacetic acid, DCC/DMAP, 68%; 2. Dess–Martin periodinane 89%. ii: 1. cinnamoyl chloride, iPr_2_NEt, 94%; 2. Dess–Martin periodinane, 86%. iii: K_2_CO_3_/MeOH).

After having demonstrated that articulated rods can be irreversibly fixed in the folded conformation we investigated the equilibrium between the straight and the folded shape of the articulated rods (cf. Figure 2). For this purpose we first chose pyrene moieties which are perfectly suited to detect this equilibrium owing to the distance dependent change in the fluorescence spectrum. Furthermore, we were interested in functional groups in the terminal positions of the ARs enabling reversible switching between the two states. This was supposed to be accomplished both by [2 + 2] photocycloaddition of derivatives of cinnamic acid and [4 + 4] photocycloaddition of anthracene derivatives. At first we pursued a sequential approach by introducing the pyrene-1-ylacetyl and the cinnamoyl moieties in a two-step sequence in 4-hydroxypiperidine with subsequent oxidation to give piperidin-4-ones **27a**,**b**. An appropriate anthracene derivative was not accessible by this approach due to the high sensitivity of the anthracene moiety against various oxidation conditions. For the construction of articulated rods we also prepared alkyne **28** by deprotection of diol **6**. In addition we used the literature known building blocks **27c** (see below the convergent approach) and **29** [9] (Scheme 6).

**Scheme 7:**
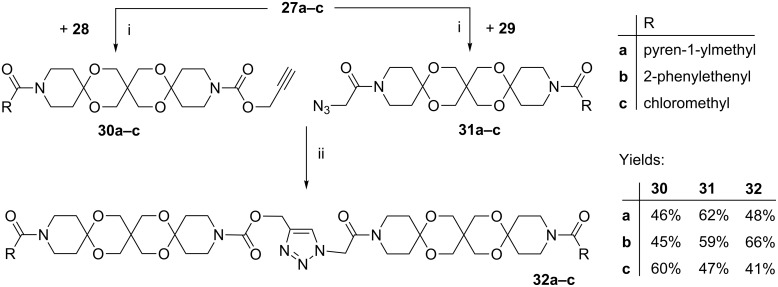
Synthesis of articulated rods **32a–c** (i: NaH, TMSCl, TMSOTf. ii: Cu/C, Et_3_N).

Based on ketones **27a**–**c** and diols **28**, **29** we obtained ARs **32a**–**c** in two steps consisting of the established double activation acetalization [8] and the CuAAC, catalyzed by Cu/C, with moderate yields (Scheme 7).

As mentioned above this sequential approach failed in the case of anthracene. In addition a convergent approach in which the terminal functionalities are introduced as late as possible in the synthesis (ideally in the last step) would be an advantage.

**Scheme 8:**
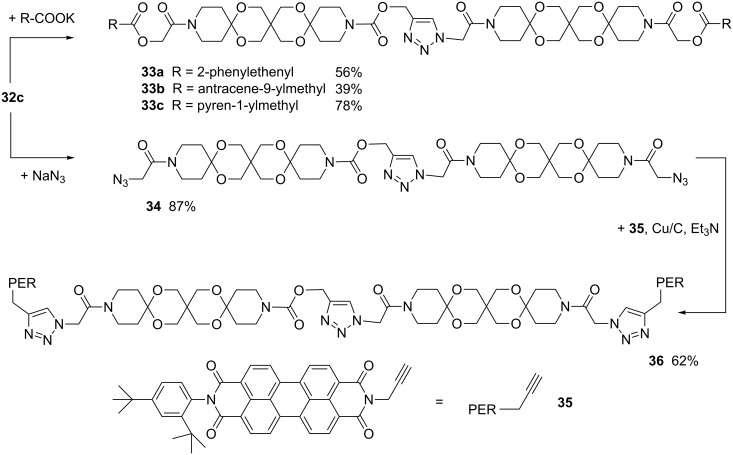
Synthesis of articulated rods **33**, **34** and **36**.

We achieved this goal starting with the ω,ω’-dielectrophilic AR **32c**. Thus, treatment with different potassium carboxylates affords compounds **33a–c** in moderate to good yields, whereas the reaction with NaN_3_ provided the ω,ω’-diazide **34**. The latter compound served as interface for labelling with various alkyne-functionalized compounds. As proof-of-concept we successfully coupled **34** with the perylene derivative **35** to give **36** (Scheme 8).

**Scheme 9:**
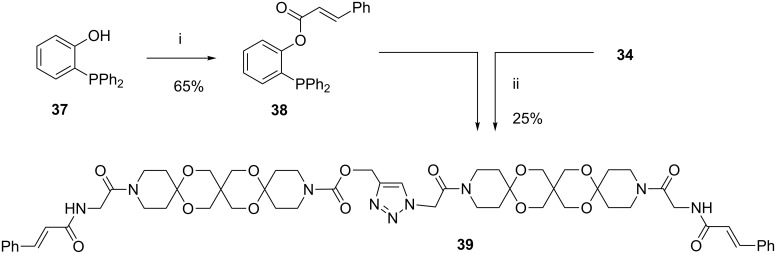
Synthesis of articulated rod **39** (i: cinnamoyl chloride, DMAP, pyridine. ii: DMF 120 °C).

Besides the widely used CuAAC ligation the Staudinger ligation [22] is also an important tool for coupling molecules. To verify that the diazide **34** is also suitable for this reaction we first prepared the cinnamoyl substituted phosphane **38** from (2-hydroxyphenyl)diphenylphosphane (**37**). By heating **34** and **38** in DMF for 3 h the AR **39** was obtained as result of a traceless Staudinger ligation, albeit with low yield (Scheme 9).

As already mentioned, we were interested in articulated rods whose folded-stretched equilibrium could be influenced by external stimuli. In order to realize this aim we chose metal cations as triggers and imidazole moieties as chelating groups at the ARs. The chelating event should be monitored by the influence on the [4 + 4] cycloaddition of anthracene.

**Scheme 10:**
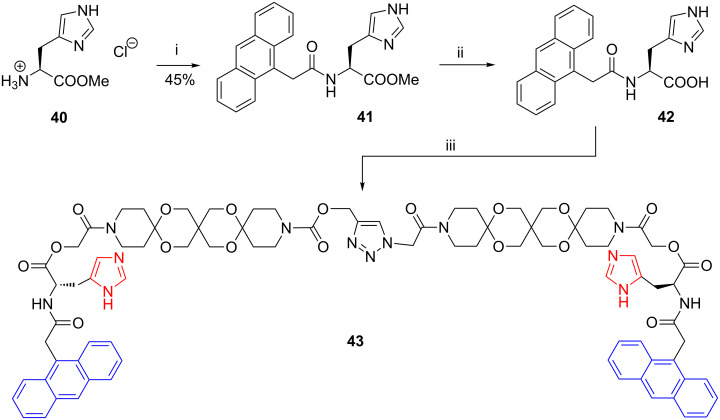
Synthesis of functionalized articulated rod **43** (i: PYBOP, Et_3_N. ii: KOH, H_2_O. iii: **32c**, quant.).

The synthesis of such a fourfold functionalized AR is based on the natural amino acid L-histidine. In the first step histidine methylester hydrochloride (**40**) is acylated with anthracene-9-acetic acid to give **41**. After saponification of the ester the potassium salt of the corresponding acid **42** was treated with **32c** affording the articulated rod **43** in quantitative yield (Scheme 10).

### The stretched–folded equilibrium of articulated rods

**Scheme 11:**
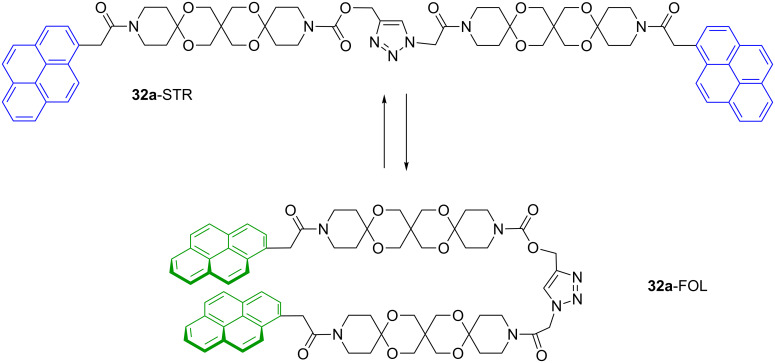
Stretched-folded equilibrium of pyrene labelled AR **32a**.

The fundamental feature of articulated rods is the restricted conformational space with two dominating species: a stretched and a folded conformation. This feature was concluded from Molecular Dynamics (MD) simulation revealing a bimodal distribution of the end-to-end distance of a model articulated rod. One maximum is located around 17 Å and can be attributed to the stretched conformation, whereas a second maximum around 7 Å corresponds to the folded species (for details see Supporting Information File 1). The investigation and the manipulation of the equilibrium between these types should therefore facilitate very interesting applications for ARs. As mentioned above, compound **32a** can serve as a probe for this equilibrium owing to the pyrene moieties. As many polyaromatic hydrocarbons pyrene possess a marked tendency to form excimers in the excited state [23]. The broad and structureless excimer emission at ca. 477 nm is red-shifted by more than 100 nm against the structured monomer emission at ca. 377 nm. This conspicuous change in the fluorescence spectra permits the investigation of the equilibrium between **32a**-STR and **32a**-FOL (Scheme 11).

**Figure 4:**
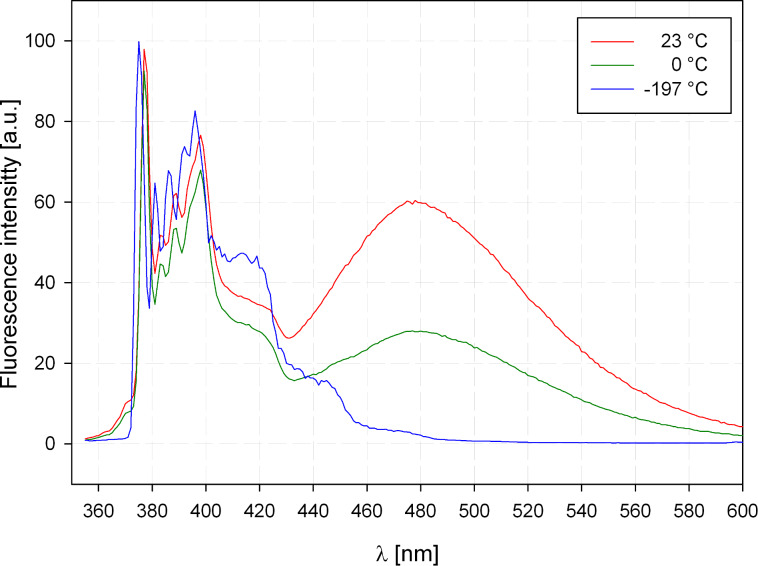
Fluorescence spectra of AR **32a** in EPA at different temperatures (*c* = 5·10^−6^ mol/L).

At first we investigated the influence of temperature and solvent viscosity on the STR–FOL equilibrium. At room temperature a ratio between the monomer and excimer emission *I*_M_/*I*_EX_ of about 100:60 is observed (Figure 4, red curve). The ratio does not change on dilution (1.5·10^−5^ mol/L → 1.1·10^−6^ mol/L) indicating that excimer formation actually proceeds intramolecularly [24]. It should be noted that the ratio *I*_M_/*I*_EX_ is not identical to the STR:FOL ratio because pyrene forms dynamic excimers, i.e., the excimer formation takes place after the excitation and there is a considerable thermodynamic driving force for the formation.

**Figure 5:**
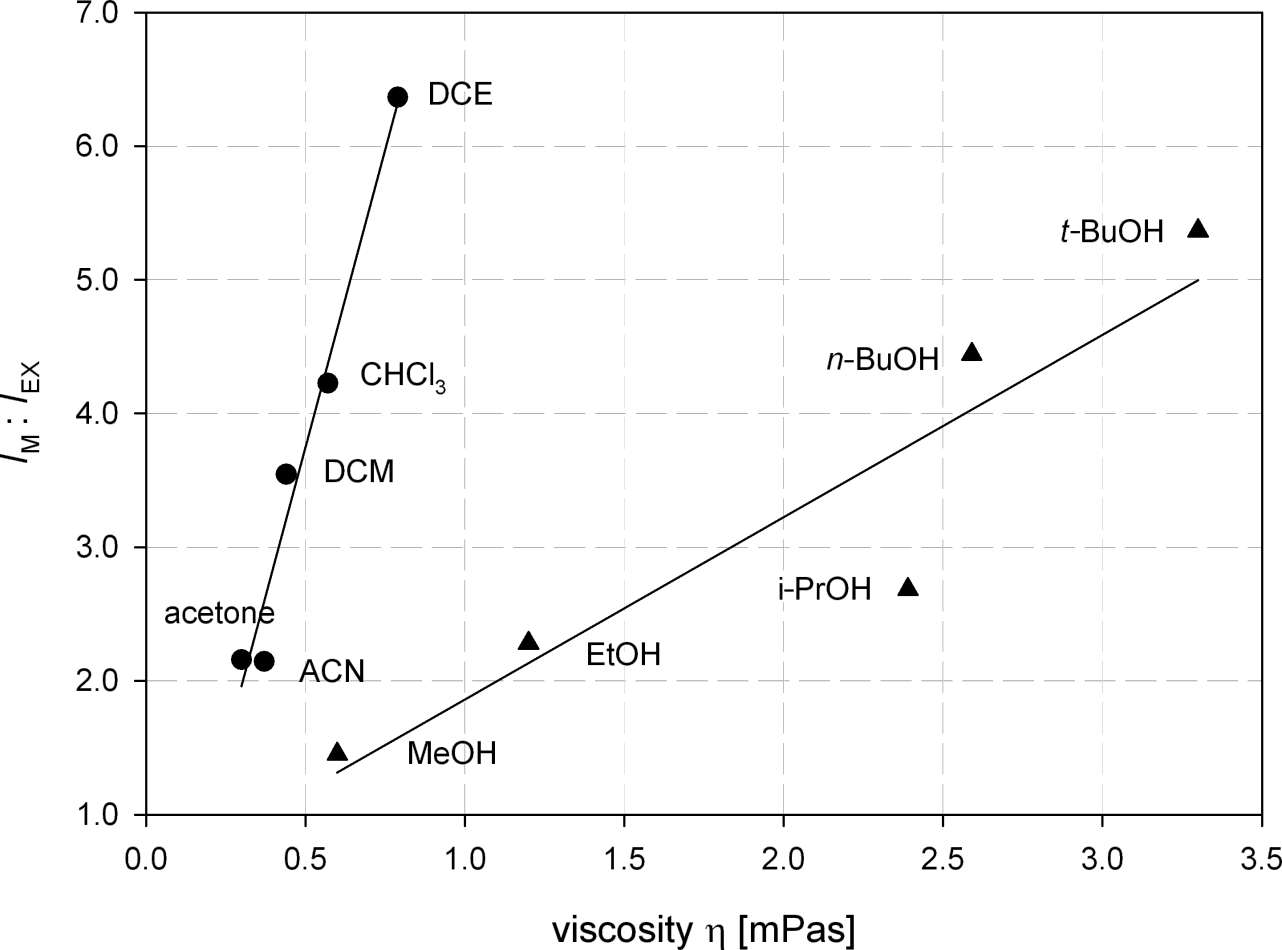
Monomer–excimer ratio *I*_M_/*I*_EX_ of the fluorescence of **32a** depending on solvent viscosity (DCE = 1,2-dichloroethane, DCM = dichloromethane, ACN = acetonitrile).

On cooling to 0 °C the *I*_M_/*I*_EX_ ratio is increased to 100:30 (Figure 4, green curve) and at 77 K in a solid EPA [25] matrix the excimer emission completely disappears (Figure 4, blue curve). This behavior underlines the above-mentioned dynamic character of the excimer and suggests that the conformer **32a**-STR is the global minimum. Based on these results we next investigated the *I*_M_/*I*_EX_ ratio in a variety of solvents with different viscosity [26]. In Figure 5 the *I*_M_/*I*_EX_ ratio is represented in dependence on the viscosity η. Two nearly linear correlations are discernable. The first group (circles) with a steep slope of the correlation line corresponds to aprotic solvents, whereas the second group (triangles) with a significantly lower slope is formed by protic solvents. Obviously protic solvents substantially stabilize the folded conformation **32a**-FOL. This can be understood by considering the rather hydrophobic backbone of the ARs and the markedly smaller surface of the FOL conformation. This behavior resembles folding of peptides and self-assembly of lipids.

**Figure 6:**
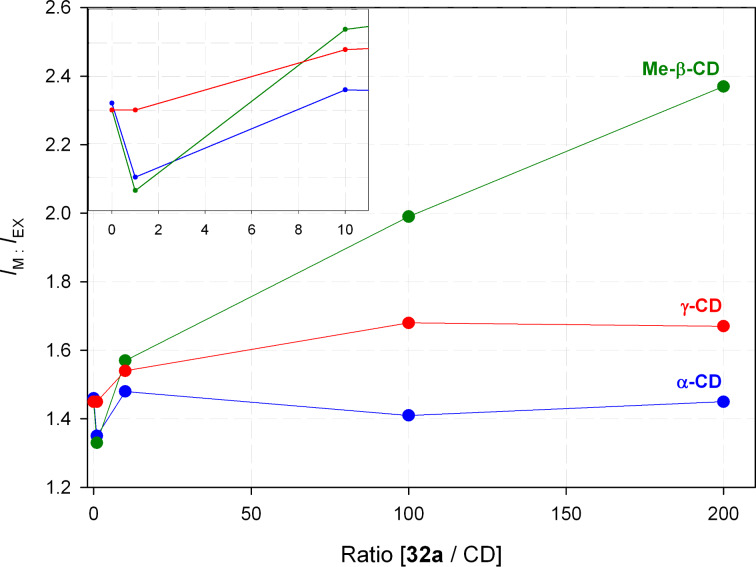
Monomer–excimer ratio *I*_M_/*I*_EX_ of the fluorescence of **32a** depending on the addition of cyclodextrines in MeOH (inset: low ratios **32a**/CD).

**Scheme 12:**
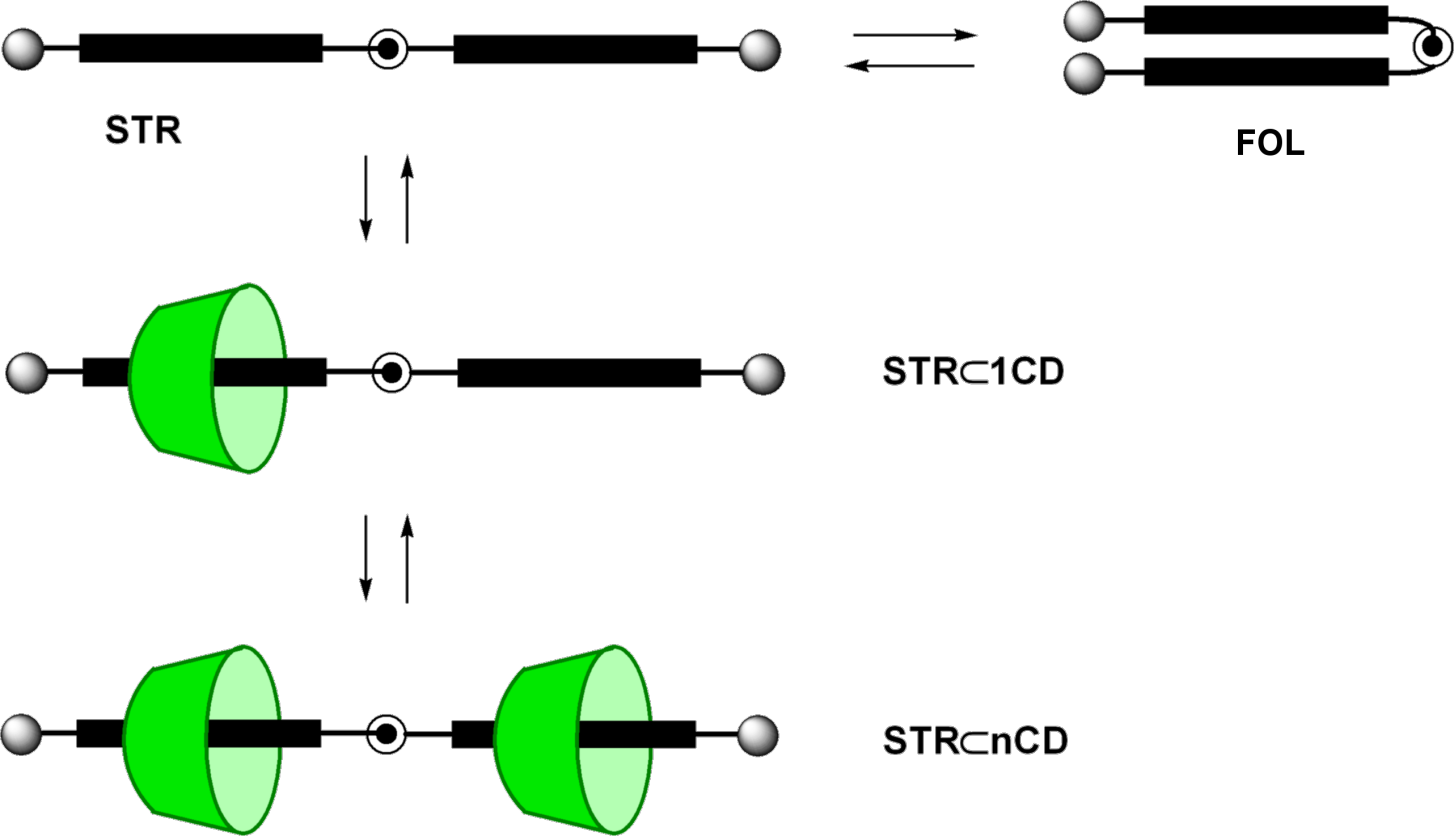
Formation of pseudorotaxanes from AR **32a** and cyclodextrines.

It is well known that cyclodextrines (CDs), bearing a hydrophobic cavity and a hydrophilic exterior, are able to encapsulate small hydrophobic molecules [27]. Therefore we hypothesized that CDs, which are threaded on articulated rods and thus form pseudorotaxanes [28], could influence the STR/FOL equilibrium, because the folding should be impaired (Scheme 12). Furthermore, we expected that the effect should depend on the size of the cavity of the CDs. As expected, the addition of Me-β-CD (which was used instead of β-CD due to the scarce solubility of the latter) in MeOH clearly increases the *I*_M_/*I*_EX_ ratio (Figure 6, green curve) whereas α- and γ-CD (blue and red curve) have hardly any influence on the fluorescence spectra of **32a**. Remarkably, low ratios of **32a** and α- or Me-β-CD, respectively, initially cause a slight decrease of the *I*_M_/*I*_EX_ ratio (Figure 6, inset). The reason for this behavior remains still unclear.

**Figure 7:**
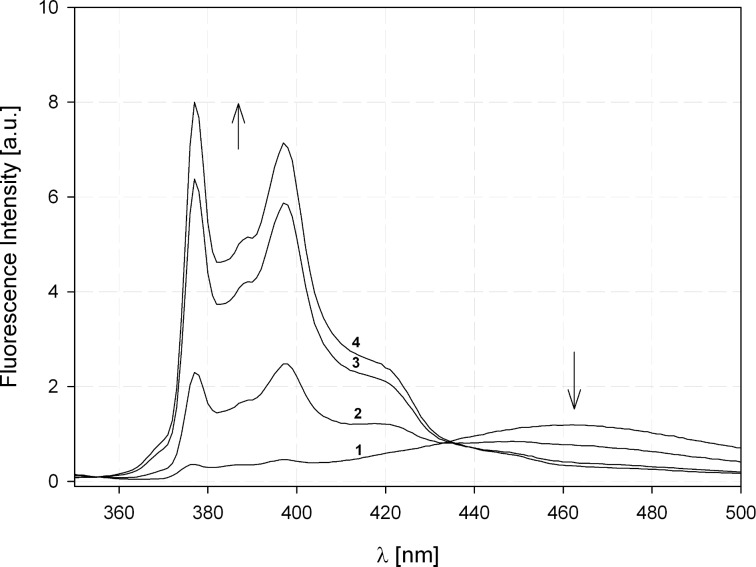
Influence of Triton X-100 on the fluorescence spectra of **32a** in aqueous solution. **32a** was added from a stock solution in MeOH to HBS giving a concentration of 2 µM. The fluorescence spectra (λ_ex_ = 340 nm) were recorded in the absence (line 1) or in the presence of Triton X-100 at 0.007 % (w/v) (line 2); 0.013 % (w/v) (line 3) and 0.02 % (w/v) (line 4) at 37 °C.

Recently we demonstrated that OSK rods are easily incorporated in biological membranes [11–13]. Because articulated rods are easier synthetically accessible, especially with different groups at each end, and remarkably better soluble, we also investigated the interaction of ARs with micelles and artificial membranes. In aqueous environments the folded conformation of **32a** predominates (indicated by an *I*_M_/*I*_EX_-ratio < 1, see Figure 7).

Upon addition of the non-ionic surfactant Triton X-100 with concentrations above the critical micelle concentration (*c*_cmc_ = 0.24 mM [29]), the signal of the excimer emission completely disappears, pointing to an incorporation of the AR in micelles in the straight conformation (Figure 7).

A similar behaviour is observed in the presence of the double-stranded lipid DOPC, which typically forms vesicles with membrane double layers. To determine the orientation of **32a** in the membranes quenching experiments with KI were undertaken (for details see Supporting Information File 1). We found a rapid decrease of the pyrene fluorescence to ca. 50% at low KI concentrations whereas considerably higher KI concentrations were required to quench the fluorescence below 50%. This outcome clearly indicates that the AR is incorporated with a perpendicular orientation to the membrane surface. Finally, we investigated the photochemical behaviour of bis-cinnamoyl substituted ARs **32b**, **33a** and **39** as well as of bis-anthracenyl substituted ARs **33b** and **43**. Herein we will only present preliminary orienting results, because the comprehensive investigation of these photoreactive systems goes beyond the scope of the present study and will be published elsewhere.

**Figure 8:**
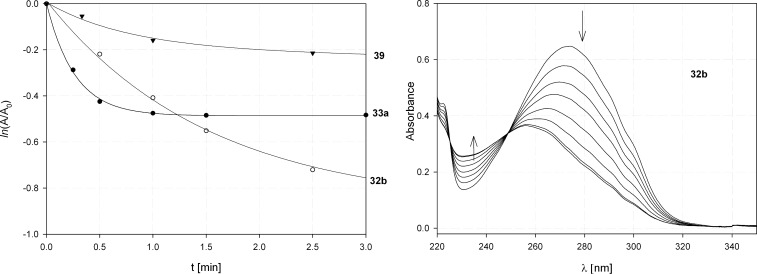
Comparison of photochemical reactivity of **32b**, **33a**, **39** (left). Irradiation UV spectrum of **32b** in ACN (right, *t* = 0, 0.5, 1, 1.5, 2.5, 4, 8, 16 min).

All of the cinnamoyl derivatives **32b**, **33a** and **39** are photochemically reactive and both the UV irradiation spectra as well as MS and NMR spectra of the crude product mixture suggest that the formation of cyclic truxinates took place as the result of intermolecular [2 + 2] cycloaddition. The photochemical reactivity of these three compounds, which differ from each other by the length and structure of the linker unit, varies considerably. In Figure 8 the photochemical decay curves of **32b**, **33a** and **39** as well as the irradiation UV spectrum of **32b** are depicted. It is clearly discernible that **32b** is substantially more reactive than **33a** and **39**.

The anthracene substituted articulated rods **33b** and **43** are also photochemically reactive. Remarkably, the photodepletion of **43** is distinctly accelerated in the presence of Ca^2+^ and Mg^2+^ ions indicating that the [4 + 4] cycloaddition is triggered by a complexation of these ions by the imidazole moieties.

## Conclusion

We have developed articulated rods (ARs) as a new class of molecular rods. The structure of our ARs is based on two (or more) conformationally rigid oligospiroketal (OSK) rods, which are linked by a flexible joint. The deciding feature of ARs is the equilibrium between two leading conformations: a straight (STR) and a folded (FOL) species. The joint contains a 1,2,3-triazole moiety and the synthesis was carried out by copper catalyzed alkyne/azide cycloaddition (CuAAC). To obtain ARs with arbitrary functionalities in the terminal positions we used trimethylsilyl alkynes and primary alcohols as protected alkynes and azides, respectively. Pursuing this approach we prepared a variety of ARs with different groups in terminal positions. The AR **32a**, bearing pyrene moieties turned out to be a versatile tool to investigate the STR/FOL equilibrium. We systematically investigated this phenomenon as a function of solvent viscosity, cyclodextrine addition and in the presence of lipids forming micelles and vesicles. In contrast to these noncovalent STR–FOL transitions we also reported on first results with respect to covalent fixation of the folded species by photochemical [2 + 2] and [4 + 4] cycloaddition. In summary, articulated rods have proven to be valuable alternatives to more rigid single rods. Especially the modular synthetic approach should open up many attractive applications.

## Supporting Information

File 1 Experimental procedures and compound characterization.
