# Supplementary material for: Articulated rods – a novel class of molecular rods based on oligospiroketals (OSK)
Source: Beilstein J Org Chem. 2015 Jan 16;11:74–84. doi: 10.3762/bjoc.11.11 (PMC4311589; doi:10.3762/bjoc.11.11)
Supplement: File 1 — Experimental procedures and compound characterization. [file Beilstein_J_Org_Chem-11-74-s001.pdf]

Supporting Information

for

Articulated rods – a novel class of molecular rods based on oligospiroketals (OSK)

Pablo Wessig*[1], Roswitha Merkel[1] and Peter Müller[2]

Address: [1]Institut für Chemie, Universität Potsdam, Karl-Liebknecht-Str. 24–25, D-14476 Potsdam, Germany and [2]Institut für Biologie/Molekulare Biophysik, Humboldt Universität zu Berlin, Invalidenstr. 42, D-10115 Berlin, Germany

Email: Pablo Wessig* - wessig@uni-potsdam.de

* Corresponding author

Experimental procedures and compound characterization

Abbreviations

ACN	acetonitrile
DBU	1,8-diazabicycloundec-7-ene
DCC	dicyclohexylcarbodimide
DCM	dichloromethane
DDQ	2,3-dichloro-5,6-dicyano-1,4-benzoquinone
DIEA	*N*,*N*-diisopropylethylamine
DMA	*N*,*N*-dimethylacetamide
DMAP	4-(dimethylamino)pyridine
DMF	*N*,*N*-dimethylformamide
DMP	Dess–Martin periodinane
DMSO	dimethyl sulfoxide
EE	ethyl acetate
FC	flash chromatography
HOBT	hydroxybenzotriazole
HV	high vacuum
m.p.	melting point

PYBOP benzotriazol-1-yloxytripyrrolidinophosphonium hexafluorophosphate
r.t. room temperature

General

DM washing solution

250 g $Na_2S_2O_3$ were dissolved in 600 mL dest. water. $NaHCO_3$ was added until saturation. The solution was filled up to 1000 ml.

pH 7 buffer

Solution A: 9.078 g KH_2PO_4 were dissolved in 1L dest. water. Solution B: 11.876 g Na_2HPO_4 were dissolved in 1 L dest. water. The ph7 buffer solution consists of 61.2 mL solution A and 38.8 mL solution B.

Cu/C catalyst

The catalyst was prepared according to ref. [1]

Synthetic Procedures

3-(Trimethylsilyl)-prop-2-ynyl 4-hydroxypiperidin-1-carboxylate (2)

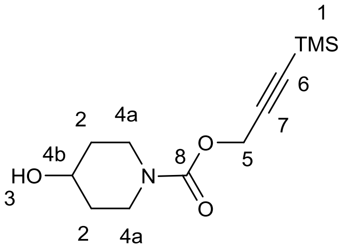

2.14 g (21.14 mmol) 4-Hydroxypiperidine were dissolved in 250 mL dry DCM. The solution was cooled (ice bath) and 6.2 g (21.14 mmol) trimethylsilylpropargyl 4-nitrophenylcarbonate were added. Upon stirring over night the solution turned yellow and a precipitate of 4-nitrophenol was formed. The mixture was concentrated and filtered over Celite®. After FC (DCM/MeOH 100/2) 4.65 g (18.21 mmol, 86%) of compound **2** were obtained as pale yellow oil. R*f* (DCM/MeOH 100/4) = 0.22; ^{1}H-NMR (300 MHz, $CDCl_3$, ppm): δ 0.16 (s, 9H, H-1), 1.46 (m, 2H, H-2), 1.76 (s, 1H, H-3), 1.83 (m, 2H, H-2), 3.13 (m, 2H, H-4a), 3.84 (m, 3H, H-4a, H-4b), 4.67 (s, 2H, H-5); ^{13}C-NMR (75.5 MHz, $CDCl_3$, ppm): δ 0.3 (C-1), 33.9 (C-2), 41.3 (C-2), 53.7 (C-5), 67.1 (C-4b), 91.5 (C-6), 99.9 (C-7), 154.4(C-8) ; IR: 2949, 2184,

1682, 1471, 1270, 1220, 1074, 1026, 839, 760 cm^{-1}; HRMS (ESI): calcd. for $C_{12}H_{22}NO_3Si$ $[M+H]^+$: 256.1369, found: 256.1356.

3-(Trimethylsilyl)prop-2-ynyl-piperidin-4-one 1-carboxylate (3)

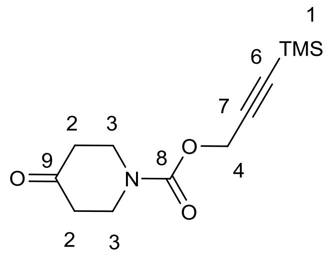

4.6 g (18.01 mmol) **2** were dissolved in 350 mL dry DCM. After cooling (ice bath) 7.64 g (18.01 mmol) DMP were added slowly. After 30 min a white precipitate has been formed. After additional 1.5 h at r.t. the solution was washed 7 times with DM-washing solution and once with brine. After drying with $MgSO_4$, evaporation and FC (DCM/MeOH 100/1) 3.89 g (15.38 mmol, 85%) of compound **3** were obtained as colorless oil. R*f* (DCM/MeOH 100/2) = 0.27; ^{1}H-NMR (300 MHz, $CDCl_3$, ppm): δ 0.18 (s, 9H, H-1), 2.36 (t, ^3J = 6 Hz, 4H, H-2), 3.78 (t, ^3J = 6 Hz, 4H, H-3), 4.74 (s, 2H, H-4); ^{13}C-NMR (75.5 MHz, $CDCl_3$, ppm): δ -0.3 (C-1), 41.0 (C-2), 43.2 (C-3), 54.2 (C-4), 92.1 (C-6), 99.4 (C-7), 154.4 (C-8), 207.0 (C-9) ; IR: 2961, 2183, 1694, 1473, 1271, 1249, 1067, 859, 762 cm^{-1}; HRMS (ESI): calcd. $C_{12}H_{20}NO_3Si$ $[M+H]^+$: 254.1212, found 254.1219.

1-(2-((4-Methoxybenzyl)oxy)acetyl)piperidin-4-ol (4)

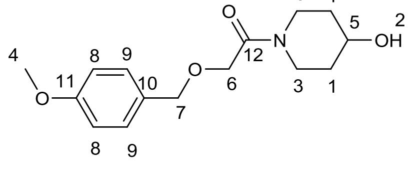

7.10 g (36.18 mmol) 2-(4-methoxybenzyloxy)acetic acid, 4.48 g (33.16 mmol) HOBT und 3,05 g (30.15 mmol) 4-hydroxypiperidine were dissolved in 150 mL dry DMF. After 30 min stirring 6.84 g (33.15 mmol) DCC were added. The mixture was stirred over night, whereby a precipitate is formed. The solid was filtered off and washed with 100 mL EE. After evaporation the orange oil was dissolved in 180 mL EE and washed with 135 mL 0.01 M HCl, 150 mL sat. $NaHCO_3$ solution and 100 ml brine. After drying with $MgSO_4$ and evaporation the crude product was purified by FC (DCM/MeOH 100/3). 6.87 g (24.60 mmol, 63%) of compound **4** were obtained as pale yellow oil. R*f* (DCM/MeOH 100/4) =

0.11; ^{1}H-NMR (300 MHz, $CDCl_3$, ppm): δ 1.51 (m, 2H, H-1), 1.73 (s, 1H, H-2), 1.85 (m, 2H, H-2), 3.20 (m, 2H, H-3), 3.72 (m, 1H, H-3), 3.92 (s, 3H, H-4), 4.04 (m, 1H, H-5), 4.14 (m, 1H, H-3), 4.14 (s, 2H, H-6), 4.52 (s, 2H, H-7), 6.86 (d, ^{3}J = 8 Hz, 2H, H-8), 7.26 (d, ^{3}J = 8 Hz, 2H, H-9); ^{13}C-NMR (75.5 MHz, $CDCl_3$, ppm): δ 33.9 (C-1), 34.5 (C-1), 42.3 (C-3), 55.3 (C-4), 67.0 (C-5), 69.3 (C-6), 72.8 (C-7), 113.9 (C-8), 129.4 (C-10), 129.7 (C-9), 159.5 (C-11), 167.7 (C-12); IR: 3391, 2934, 2861, 1618, 1512, 1448, 1245, 1075, 1028, 818 cm^{-1}; HRMS (ESI): calcd. for $C_{15}H_{22}NO_4$ $[M+H]^+$: 280.1549, found 280.1536.

1-(2-((4-Methoxybenzyl)oxy)acetyl)piperidin-4-one (5)

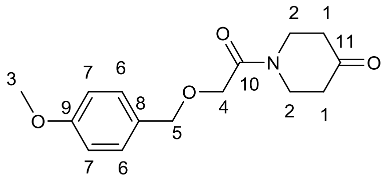

90 mL dry DCM were cooled to -60 °C. 2.39 mL (33.67 mmol) dry DMSO und 2.15 mL (25.26 mmol) oxalylchloride were successively added drop-wise. The mixture was stirred 45 min and then 4.70 g (16.84 mmol) of compound **4**, dissolved in 10 mL DCM were added slowly. After additional stirring for 45 min 12.17 mL (84.18 mmol) dry Et_3N were added and the white suspension was allowed to warm up to r.t. It was stirred for 1h and then washed twice with aqueous tartaric acid solution (20%, 40 mL, 20 mL). The solution was dried with $MgSO_4$, evaporated and the residue was purified by FC (DCM/MeOH 100/2). 4.04 g (14.55 mmol, 86%) **5** were obtained as white solid. R*f* (DCM/MeOH 100:2) = 0.13 ; ^{1}H-NMR (300 MHz, $CDCl_3$, ppm): δ 2.44 (m, 4H, H-1), 3.75 (m, 2H, H-2), 3.80 (s, 3H, H-3), 3.85 (m, 2H, H-2), 4.21 (s, 2H, H-4), 4.54 (s, 2H, H-5), 6.86 (d, ^{3}J = 8 Hz, 2H, H-6), 7.25 (d, ^{3}J = 8 Hz, 2H, H-7); ^{13}C-NMR (75.5 MHz, $CDCl_3$, ppm): δ 40.8 (C-1), 41.4 (C-1), 43.9 (C-2), 55.3 (C-3), 69.6 (C-4), 73.15 (C-5), 113.9 (C-7), 129.0 (C-8), 129.7 (C-6), 159.6 (C-9), 168.1 (C-10), 206.6 (C-11); IR: 2934, 2868, 1714, 1646, 1611, 1512, 1446, 1243, 1082, 1030, 819 cm^{-1}; HRMS (ESI): calcd. for $C_{15}H_{20}NO_4$ $[M+H]^+$: 278.1392, found 278.1392.

3-(Trimethylsilyl)prop-2-ynyl 3,3-bis(hydroxymethyl)-1,5-dioxa-9-azaspiro[5.5]undecane-9-carboxylate (6)

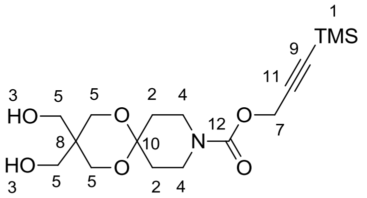

2.57 g (10,11 mmol) **3**, 2.07 g (15,2 mmol) pentaerythritol und 96.5 mg (506,77 µmol) *p*-toluenesulfonic acid were added to 85 mL of a benzene/DMF mixture (3:2). The mixture was stirred at 140°C (bath temperature) using a Dean-Stark trap. The solution was cooled to r.t., neutralized with 45 mg (506.77 µmol) solid $NaHCO_3$ and evaporated (rotary evaporator, then HV). After purification by FC (DCM/MeOH 100:4) 2.74 g (7.39 mmol, 73%) **6** were obtained as white solid. R*f* (DCM/MeOH 100:4) = 0.11 ; ^{1}H-NMR (300 MHz, $CDCl_3$, ppm): δ 0.17 (s, 9H, H-1), 1.83 (s, 4H, H-2), 2.62 (m, 2H, H-3), 3.50 (t, ^3J = 6 Hz, 4H, H-4), 3.73 (s, 8H, H-5), 4.69 (s, 2H, H-7); ^{13}C-NMR (75.5 MHz, $CDCl_3$, ppm): δ -0.3 (C-1), 32.0 (C-2), 32.6 (C-2), 39.3 (C-8), 40.8 (C-4), 53.8 (C-7), 62.1 (C-5), 64.7 (C-5), 91.6 (C-9), 96.7 (C-10), 99.8 (C-11), 154.4 (C-12); Fp = 121.5-123 °C; IR: 2963, 2865, 2184, 1692, 1470, 1433, 1357, 1275, 1247, 1232, 1113, 1068, 1028, 840 cm^{-1}; HRMS (ESI): calcd. for $C_{17}H_{30}NO_6Si$ $[M+H]^+$: 372.1842, found 372.1852.

3-(Trimethylsilyl)prop-2-yn-1-yl 15-{[(4-methoxybenzyl)oxy]acetyl}-7,11,18,21-tetraoxa-3,15-diazatrispiro[5.2.2.5^{12}.2^9.2^6]heneicosane 3-carboxylate (7)

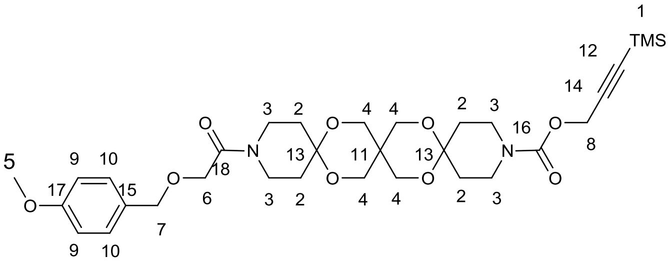

3.9 g (14.1 mmol) of ketone **5** were suspended in 450 mL dry Et_2O and cooled to 0°C. After addition of 564 mg (14,10 mmol) NaH (60 % content) and 1.8 mL (14.10 mmol) TMSCl the suspension was stirred for 1h at r.t. Then, 5.24 g (14.10 mmol) of diol **6** und one drop TMSOTf were added and the mixture was stirred over night. The precipitated product **7** was filtered off to give 6.87 g (10.90 mmol, 77%) as white solid. R*f* ($CHCl_3$/EE 2/1) = 0.30; ^{1}H-NMR (300 MHz, $CDCl_3$, ppm): δ 0.12 (s, 9H, H-1), 1.76 (m, 8H, H-2), 3.39 (m, 2H, H-3), 3.44 (m, 4H, H-3), 3.54 (m, 2H, H-3), 3.68 (t, ^3J = 10 Hz, 8H, H-4), 3.73 (s, 3H, H-5), 4.07 (s, 2H, H-6), 4.44 (s, 2H, H-7), 4.63 (s, 2H, H-8), 6.80 (d, ^3J = 9 Hz, 2H, H-9),

7.19 (d, ^3J = 9 Hz, 2H, H-10); ^{13}C-NMR (75.5 MHz, $CDCl_3$, ppm): δ 0.1 (C-1), 31.7 (C-2), 33.5 (C-11), 33.9 (C-2), 39.0 (C-3) , 41.2 (C-3), 42.1 (C-3), 54.1 (C-8), 55.6 (C-5), 63.8 (C-4), 69.6 (C-6), 73.2 (C-7), 91.9 (C-12), 97.3 (C-13), 100.3 (C-14), 114.2 (C-10), 129.7 (C-15), 130.0 (C-9), 154.7 (C-16), 159.8 (C-17), 168.0 (C-17); m.p. 96-100 °C; IR: 2959, 2866, 2183, 1706, 1653, 1610, 1511, 1468, 1437, 1360, 1345, 1248, 1228, 1095, 1036, 846cm^{-1}; HRMS (ESI): calcd. for $C_{32}H_{46}N_2O_9NaSi$ $[M+Na]^+$: 653.1870, found 653.2815.

3-(Trimethylsilyl)prop-2-yn-1-yl 15-(hydroxyacetyl)-7,11,18,21-tetraoxa-3,15-diazatrispiro[5.2.2.5^{12}.2^9.2^6]heneicosan-3-carboxylate (8)

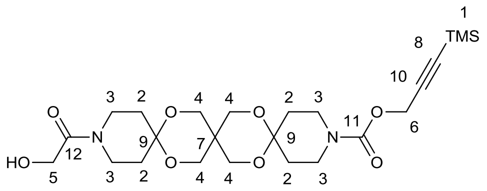

5.20 g (8.56 mmol) **7** and 9.72 g (42.80 mmol) DDQ were dissolved in 840 mL of a mixture of DCM/pH7 buffer (9:1) and stirred over night. A black aqueous and a red organic phase are formed. The phases were separated and the aqueous phase was extracted with 150 mL DCM. The combined organic phases were washed twice with 200 mL sat. aqueous $NaHCO_3$ solution and then with brine. After drying and evaporating a red oil was obtained, which purified by FC (DCM/MeOH 100:2). 3.89 g (7.62 mmol, 89%) **8** were obtained. R*f* (DCM/MeOH 100/4) = 0.29; ^{1}H-NMR (300 MHz, $CDCl_3$, ppm): δ 0.16 (s, 9H, H-1), 1.61 (m, 8H, H-2), 3.32 (m, 2H, H-3), 3.49 (m, 4H, H-3), 3.65 (m, 2H, H-3), 3.78 (m, 8H, H-4), 4.14 (s, 2H, H-5), 4.68 (s, 2H, H-6); ^{13}C-NMR (75.5 MHz, $CDCl_3$, ppm): δ -0.3 (C-1), 31.1 (C-2), 33.2 (C-7), 39.2 (C-3) , 40.1 (C-3), 40.8 (C-3), 53.7 (C-6), 59.7 (C-5), 63.5 (C-4), 91.6 (C-8), 97.0 (C-9), 99.9 (C-10), 154.3 (C-11), 169.9 (C-12); Fp = 169-171 °C; IR: 2961, 2862, 2190, 1701, 1637, 1483, 1469, 1423, 1364, 1347, 1251, 1228, 1090, 1039, 844cm^{-1}; HRMS (ESI): calcd. for $C_{24}H_{39}N_2O_8Si$ $[M+H]^+$: 511.2476, found 511.2454.

3-(Trimethylsilyl)prop-2-yn-1-yl 15-(azidoacetyl)-7,11,18,21-tetraoxa-3,15-diazatrispiro[5.2.2.5^{12}.2^{9}.2^{6}]heneicosan-3-carboxylate (9)

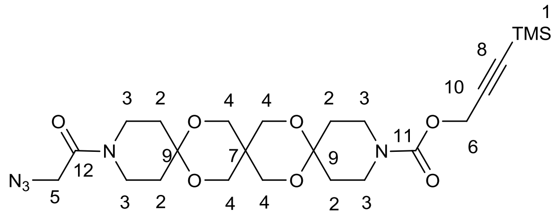

1.85 g (3.62 mmol) **8**, 353 mg (5.42 mmol) sodium azide, 1.80 g (5.43 mmol) tetrabromomethane and 1.43 g (5.43 mmol) triphenylphosphane were dissolved in 90 mL dry DMF and stirred over night. The mixture was evaporated and dried in HV. After purification with FC (DCM/MeOH 100:2) 1.37 g (2.56 mmol, 71%) **9** were obtained as white solid. R*f* (DCM/MeOH 100/3) = 0.22; ^{1}H-NMR (300 MHz, $CDCl_3$, ppm): δ 0.18 (s, 9H, H-1), 1.83 (m, 8H, H-2), 3.37 (m, 2H, H-3), 3.50 (m, 4H, H-3), 3.64 (m, 2H, H-3), 3.75 (m, 8H, H-4), 3.93 (s, 2H, H-5), 4.70 (s, 2H, H-6); ^{13}C-NMR (75.5 MHz, $CDCl_3$, ppm): δ -0.3 (C-1), 31.0 (C-2), 33.1 (C-7), 33.5 (C-2), 38.9 (C-3) , 40.7 (C-3), 41.8 (C-3), 50.7 (C-5), 53.7 (C-6), 63.4 (C-4), 91.5 (C-8), 96.6 (C-9), 99.9 (C-10), 154.3 (C-11), 169.4 (C-12); m.p. 47-50 °C; IR: 2963, 2870, 2183, 2106, 1701, 1657, 1438, 1359, 1231, 1094, 1037, 847 cm^{-1}; HRMS (ESI): calcd. for $C_{24}H_{37}N_2O_7NaSi$ $[M+Na]^+$: 558.2360, found 558.2331.

Prop-2-yn-yl 15-(hydroxyacetyl)-7,11,18,21-tetraoxa-3,15-diazatrispiro[5.2.2.5^{12}.2^{9}.2^{6}]heneicosan-3-carboxylate (10)

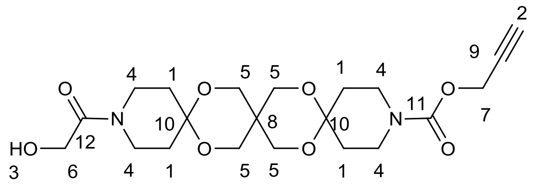

1.80 g (3.52 mmol) of compound **8** and 729 mg (4.22 mmol) K_2CO_3 were dissolved in 45 mL MeOH and stirred for 3h. 18 mL pH7 buffer were added and the mixture was extracted with twice with 130 mL DCM. The combined organic solutions were washed with brine, dried with $MgSO_4$ and evaporated. After purification by FC (DCM/MeOH 100:3) 1.43 g (3.26 mmol, 93%) **10** were obtained as white solid. R*f* (DCM/MeOH 100/5) = 0,26; 1H-NMR (300 MHz, $CDCl_3$, ppm): δ 1.85 (m, 8H, H-1), 2.24 (t, ^{3}J = 2.4 Hz, 1H, H-2), 3.24 (m, 3H, H-3, H-4), 3.48 (m, 4H, H-4), 3.64 (m, 2H, H-4), 3.73 (m, 8H, H-5), 4.13 (s, 2H, H-6), 4.66 (s, 2H, H-7); ^{13}C-NMR (75.5 MHz, $CDCl_3$, ppm): δ 31.0 (C-1), 31.5 (C-1), 33.1 (C-8), 39.1 (C-4) , 40.1 (C-4), 40.7 (C-4), 52.9 (C-7), 59.6 (C-6), 63.4 (C-5), 74.4 (C-2), 78.4 (C-9), 96.6 (C-10), 96.9 (C-10), 154.2 (C-11), 169.8 (C-12); m.p. 151 °C; IR: 2959, 2869, 2119, 1698, 1630,

1471, 1440, 1381, 1362, 1269, 1232, 1164, 1096, 888 cm^{-1}; HRMS (ESI): calcd. for $C_{21}H_{30}N_2O_8Na$ $[M+Na]^+$: 461.1900, found 461.1897.

(1-{2-Oxo-2-[15-({[3-trimethylsilyl)prop-2-yn-1-yl]oxy}carbonyl)-7,11,18,21-tetraoxa-3,15-diazatrispiro[5.2.2.5^{12}.2^{9}.2^{6}]heneicos-3-yl]ethyl}-1*H*-1,2,3-triazol-4-yl)methyl-15-(hydroxyacetyl)-7,11,18,21-tetraoxa-3,15-diazatrispiro[5.2.2.5^{12}.2^{9}.2^{6}]heneicosan-3-carboxylate (11)

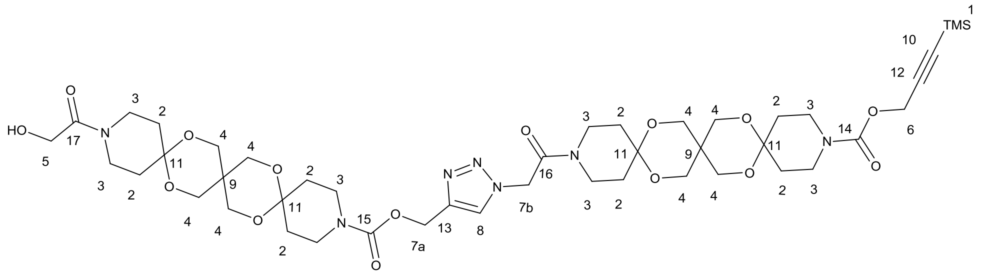

800 mg (1.82 mmol) **10** and 987 mg (1.84 mmol) **9** were dissolved in 3.6 mL DCM/MeOH mixture (1:1). 260 µL (1.88 mmol) Et_3N and 73 mg Cu/C catalyst (1.01 mmol/g) were added and the mixture was stirred over night. After evaporation the crude product was purified by FC (DCM/MeOH 100:5) to give 1.45 g (1.49 mmol, 82%) **11** as white solid. R*f* (DCM/MeOH 100:5) = 0.18; ^{1}H-NMR (300 MHz, $CDCl_3$, ppm): δ 0.17 (s, 9H, H-1), 1.83 (m, 16H, H-2), 3.24 (m, 2H, H-3), 3.47 (m, 10H, H-3), 3.69 (m, 20H, H-3, H-4), 4.14 (s, 2H, H-5), 4.68 (s, 2H, H-6), 5.21 (s, 4H, H-7a,H-7b), 7.79 (s, 1H, H-8); ^{13}C-NMR (75.5 MHz, $CDCl_3$, ppm): δ -0.3 (C-1), 31.1 (C-2), 33.1 (C-9), 39.2 (C-3), 40.1 (C-3), 40.6 (C-3), 42.1 (C-3), 51.0 (C-7b), 53.7 (C-6), 58.3 (C-7a) 59.6 (C-5), 63.4 (C-4), 91.5 (C-10), 96.9 (C-11), 99.8 (C-12), 125.5 (C-8), 143.6 (C-13), 154.3 (C-14), 154.8 (C-15), 163.0 (C-16), 169.8 (C-17); m.p. 127-128 °C; IR: 2962, 2866, 2183, 1697, 1653, 1470,1440, 1363, 1344, 1269, 1231, 1095, 1037, 846 cm^{-1}; HRMS (ESI): calcd. for $C_{45}H_{68}N_7O_{15}Si$ $[M+H]^+$: 974.4543, found 974.4628.

[1-(2-Oxo-2-{15-[(prop-2-yn-1-yloxy)carbonyl]-7,11,18,21-tetraoxa-3,15-diazatrispiro[5.2.2.5^{12}.2^{9}.2^{6}]heneicos-3-yl}ethyl)-1*H*-1,2,3-triazol-4-yl]methyl-15-(hydroxyacetyl)-7,11,18,21-tetraoxa-3,15-diazatrispiro[5.2.2.5^{12}.2^{9}.2^{6}]heneicosan-3-carboxylate (12)

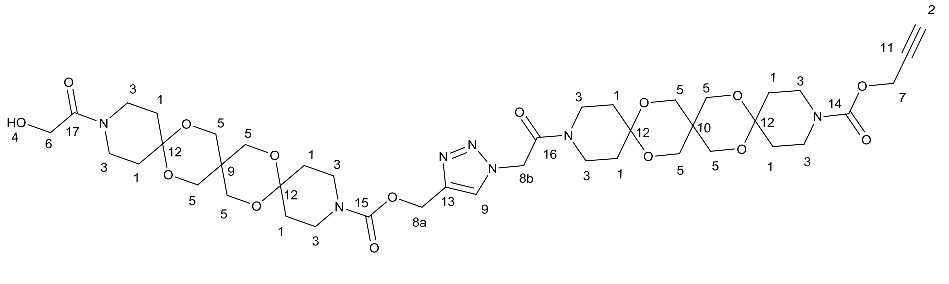

503 mg (516.77 µmol) **11** were dissolved in 13 mL MeOH and 142 mg (1.03 mmol) K_2CO_3 were added. The turbid solution became clear and a new precipitate was formed. After 2h 5 mL pH7 buffer was added and was evaporated. After purification by FC (DCM/MeOH 100:3) 406 mg (450.04 µmol, 87%) **12** were obtained as white solid. R*f* (DCM/MeOH 100/7) = 0.31; ^{1}H-NMR (300 MHz, $CDCl_3$, ppm): δ 1.81 (m, 16H, H-1), 2.45 (t, ^3J = 2.4 Hz, 1H, H-2), 3.23 (m, 2H, H-3), 3.48 (m, 11H, H-3, H-4), 3.63 (m, 4H, H-3), 3.72 (m, 16H, H-5), 4.13 (s, 2H, H-6), 4.66 (s, 2H, H-7), 5.21 (m, 4H, H-8a, H-8b), 7.78 (s, 1H, H-9); ^{13}C-NMR (75.5 MHz, $CDCl_3$, ppm): δ 31.0 (C-1), 31.1 (C-1), 32.9 (C-10), 39.0 (C-3), 40.5 (C-3), 40.6 (C-3), 41.9 (C-3), 50.8 (C-8b), 52.8 (C-7), 58.3 (H-8a), 59.5 (C-6), 63.1 (C-5), 63.3 (C-5), 74.4 (C-2), 78.4 (C-11), 96.3 (C-12), 96.5 (C-12), 96.7 (C-12), 125.3 (C-9), 143.5 (C-13), 154.0 (C-14), 154.7 (C-15), 163.0 (C-16), 169.7 (C-17); m.p. 125-126 °C; IR (in $CHCl_3$): 3013, 2940, 2869, 2123, 1695, 1654, 1472, 1441, 1362, 1274, 1233, 1166, 1092, 891cm^{-1}; HRMS (ESI): calcd for $C_{42}H_{60}N_7O_{15}$ [M+H]$^+$: 902.4147, found 902.4119.

(1-{2-Oxo-2-[15-({[3-trimethylsilyl)prop-2-yn-1-yl]oxy}carbonyl)-7,11,18,21-tetraoxa-3,15-diazatrispiro[5.2.2.5^{12}.2^9.2^6]heneicos-3-yl]ethyl}-1*H*-1,2,3-triazol-4-yl)methyl-15-(azidoacetyl)-7,11,18,21-tetraoxa-3,15-diazatrispiro[5.2.2.5^{12}.2^9.2^6]heneicosan-3-carboxylate (13)

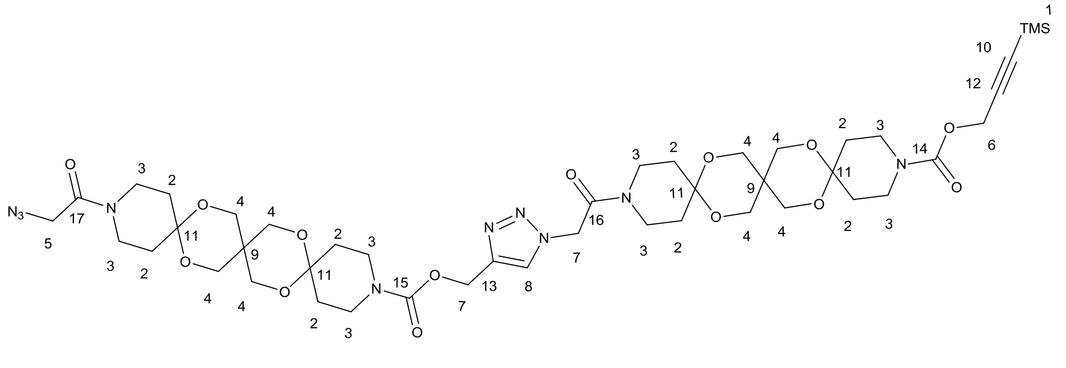

250 mg (256.64 µmol) **11**, 50 mg (769.11 µmol) sodium azide, 128 mg (386.01 µmol) tetrabromo-methane and 101 mg (386.01 µmol) triphenylphosphane were dissolved in 7 mL dry DMF and stirred over night. The solution was evaporated, dried in HV and the residue was purified by FC (DCM/MeOH 100:5) to give 139 mg (139.14 µmol, 54%) **13** as white solid. R*f* (DCM/MeOH 100:7) = 0.36; ^{1}H-NMR (300 MHz, $CDCl_3$, ppm): δ 0.17 (s, 9H, H-1), 1.83 (m, 16H, H-2), 3.36 (m, 2H, H-3), 3.48 (m, 10H, H-3), 3.62 (m, 4H, H-3), 3.73 (m, 16H, H-4), 3.92 (s, 2H, H-5), 4.69 (s, 2H, H-6), 5.21 (s, 4H, H-7), 7.79 (s, 1H, H-8); ^{13}C-NMR (75.5 MHz, $CDCl_3$, ppm): δ -0.3 (C-1), 29.6 (C-2), 31.0 (C-2), 33.1 (C-9), 33.6 (C-2), 39.2 (C-3), 40.7 (C-3), 41.8 (C-3), 50.7 (C-7), 51.0 (C-5), 53.8 (C-6), 58.3 (C-7), 63.4 (C-4), 91.6 (C-10), 96.9 (C-11), 99.8 (C-12), 125.5 (C-8), 143.6 (C-13), 154.3 (C-14), 154.9 (C-15), 163.0 (C-16), 165.4 (C-17);

m.p. 115-120 °C; IR: 2968, 2777, 2106, 1698, 1537, 1468, 1439, 1363, 1231, 1198, 1094, 1037, 889, 845 cm^{-1}; HRMS (ESI): calcd for $C_{45}H_{67}N_{10}O_{14}Si$ $[M+H]^+$: 999.4608, found 999.4656.

{1-[2-Oxo-2-(15-{[(1-{2-oxo-2-[15-({[1-(2-oxo-2{15-({[3-(trimethylsilyl)prop-2-yn-1-yl]oxy)carbonyl)-7,11,18,21-tetraoxa-3,15-diazatrispiro[5.2.2.5^{12}.2^{9}.2^{6}]heneicos-3-yl]ethyl}-1*H*-1,2,3-triazol-4-yl)methoxy]carbonyl}-7,11,18,21-tetraoxa-3,15-diazatrispiro[5.2.2.5^{12}.2^{9}.2^{6}]heneicos-3-yl)ethyl]-1H-1,2,3-triazol-4-yl}methoxy)carbonyl]-7,11,18,21-tetraoxa-3,15-diazatrispiro[5.2.2.5^{12}.2^{9}.2^{6}]heneicos-3-yl}ethyl)-1*H*-1,2,3-triazol-4-yl]methyl-15-(hydroxyacetyl)-7,11,18,21-tetraoxa-3,15-diazatrispiro[5.2.2.5^{12}.2^{9}.2^{6}] heneicosan-3-carboxylate (14)

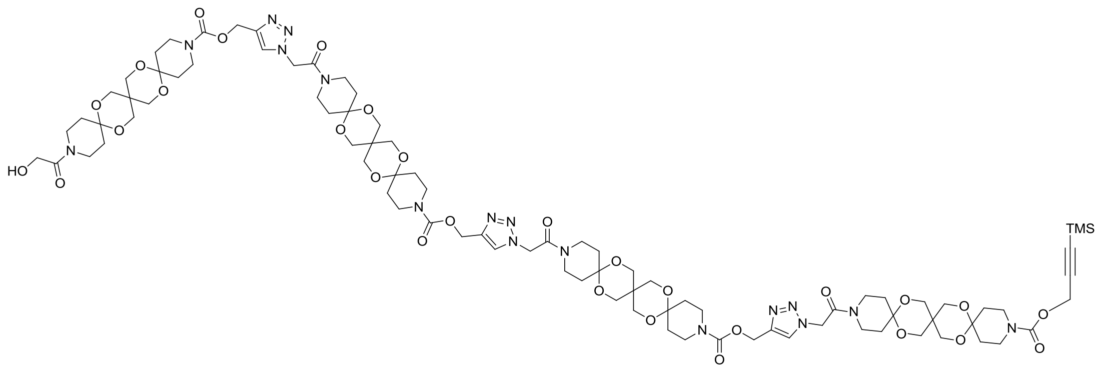

60 mg (60 µmol) **13** and 54 mg (60 µmol) **12** were dissolved in 137 µL DCM/MeOH (1:1). 8.9 µL (60 µmol) Et_3N and 64 mg Cu/C catalyst (1.01 mmol/g) were added and the mixture was stirred over night. After addition of 1M HCl the mixture was extracted twice with DCM. The combined organic phases were washed with sat. $NaHCO_3$ solution and brine, dried with $MgSO_4$ and evaporated. After precleaning with FC (DCM/MeOH 100:8) 68.44 mg (36 µmol, 60%) product **14** were isolated after preparative HPLC (mobile phase: DCM/MeOH 100:6)**.** R*f* (DCM/MeOH 100:8) = 0.38; ^{1}H-NMR (300 MHz, $CDCl_3$, ppm): δ 0.15 (s, 5,5 H, H-1a), 1.80 (m, 32H, H-2), 3.23 (m, 2H, H-3), 3.43 (m, 21H, H-3, H-4), 3.60 (m, 8H, H-3), 3.71 (m, 34H, H-3, H-4), 4.13 (s, 2H, H-6), 4.66 (s, 2H, H-7), 5.18 (m, 12H, H-8), 7.77 (s, 3H, H-9); ^{13}C-NMR (75.5 MHz, $CDCl_3$, ppm): δ -0.4 (C-1), 31.2 (C-2), 33.0 (C-10), 39.1 (C-3), 40.6 (C-3), 42.0 (C-3), 50.9 (C-8), 53.4 (C-7), 58.4 (C-8), 59.6 (C-6), 63.4 (C-5), 91.3 (C-11), 96.4 (C-12), 96.9 (C-12), 99.5 (C-13), 125.4 (C-9), 143.6 (C-14), 154.3 (C-15), 154.8 (C-16), 163.0 (C-17), 169.8 (C-18); m.p. 217-220 °C; IR: 2966, 2870, 2245, 1697, 1656, 1471,1440, 1363, 1342, 1269, 1232, 1095, 847cm^{-1}; HRMS (ESI): calcd for $C_{87}H_{126}N_{17}O_{29}Si$ $[M+H]^+$: 1900.8677, found 1900.8661.

[1-(2-Oxo-2-{15-[(prop-2-yn-1-yloxy)carbonyl]-7,11,18,21-tetraoxa-3,15-diazatrispiro[5.2.2.5^{12}.2^{9}.2^{6}]heneicos-3-yl}ethyl)-1*H*-1,2,3-triazol-4-yl]methyl-15-(azidoacetyl)-7,11,18,21-tetraoxa-3,15-diazatrispiro[5.2.2.5^{12}.2^{9}.2^{6}]heneicosan-3-carboxylate (15)

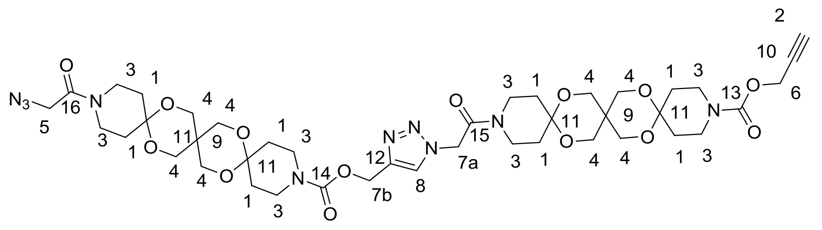

270 mg (298.99 µmol) **12**, 29.16 mg (448.50 µmol) sodium azide, 297 mg (896.98 µmol) tetrabromo-methane and 118 mg (448.50 µmol) triphenylphosphane were dissolved in 8 mL dry DMF and stirred over night. The solution was evaporated, dried in HV and the residue was purified by FC (DCM/MeOH 100:5) to give 174 mg (188.10 µmol, 63%) **15** as white solid. R*f* (DCM/MeOH 100/7) = 0.38; ^{1}H-NMR (300 MHz, $CDCl_3$, ppm): δ 1.83 (m, 16H, H-1), 2.45 (t, ^{3}J = 2.4 Hz, 1H, H-2), 3.35 (m, 2H, H-3), 3.45 (m, 10H, H-3), 3.50 (m, 4H, H-3), 3.72 (m, 16H, H-4), 3.92 (s, 2H, H-5), 4.67 (d, ^{3}J = 2.4 Hz, 2H, H-6), 5.22 (m, 4H, H-7a, H-7b), 7.79 (s, 1H, H-8); ^{13}C-NMR (75.5 MHz, $CDCl_3$, ppm): δ 31.2 (C-1), 31.3 (C-1), 33.0 (C-9), 33.0 (C-1), 38.8 (C-3), 39.1 (C-3), 40.6 (C-3), 42.0 (C-3), 50.9 (C-5), 51.3 (C-7a), 52.9 (C-6), 58.4 (H-7b), 63.3 (C-4), 74.4 (C-2), 78.4 (C-10), 96.4 (C-11), 96.8 (C-11), 125.4 (C-8), 143.5 (C-12), 154.1 (C-13), 154.8 (C-14), 163.0 (C-15), 165.3 (C-16); m.p. 155 °C; IR (in $CHCl_3$): 2965, 2871, 2362, 2107, 1695, 1658, 1471, 1442, 1363, 1274, 1232, 1166, 1094, 891 cm^{-1}; HRMS (ESI): calcd for $C_{42}H_{59}N_{10}O_{14}$ $[M+H]^+$: 927.4212, found 927.4190.

(2*S*)-1-(4-Hydroxypiperidin-1-yl)-2-[(4-methoxybenzyl)oxy]-4-methylpentan-1-one (17)

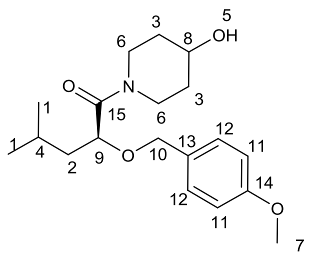

1.95 g (7.22 mmol) *S*-2-(4-Methoxybenzyloxy)-4-methylpentanoic acid [2], 730 mg (7.22 mmol) 4-hydroxypiperidine and 1.07 g (7.94 mmol) HOBT were dissolved in 16 mL dry DMF. After 30 min stirring 1.64 g (7.94 mmol) DCC were added. The mixture was stirred over night, the precipitate (dicyclohexyl urea) was filtered off and washed with EE. The combined organic phases was

evaporated and dried in HV. The residue was dissolved in 20 mL EE, was successively washed with 14 mL 0.01 M HCl, 15 mL sat. $NaHCO_3$ and 15 mL brine. After drying with $MgSO_4$ and evaporation the residue was purified by FC (DCM/MeOH 100:4) to give 2.40 g (7,16 mmol, 99%) **17** as colorless oil. R*f* (DCM/MeOH 100:4) = 0,17; ^{1}H-NMR (300 MHz, $CDCl_3$, ppm): δ 0.84 (d*d, ^3J = 24.8 Hz, ^4J = 6.5 Hz, 6H, H-1), 1.42 (m, 3H, 1*H-2, 2*H-3), 1.77 (m, 4H, 1*H-2, 2*H-3, H-4), 2.25 (s, 1H, H-5), 3.25 (m, 2H, H-6), 3.79 (s, 3H, H-7), 4.02 (m, 3H, H-6, H-8), 4.20 (d*d, ^3J = 9.6 Hz, ^4J = 4.2 Hz, 1H, H-9), 4.32 (m, 1H, H-10), 4.53 (m, 1H, H-10), 6.84 (d, ^3J = 8.7 Hz, 2H, H-11), 7.22 (d, ^3J = 8.7 Hz, 2H, H-12); ^{13}C-NMR (75.5 MHz, $CDCl_3$, ppm): δ 21.6 (C1), 23.16 (C-1), 24.7 (C-4), 34.2 (C-3), 39.6 (C-6), 41.5 (C-2), 41.9 (C-6), 55.2 (C-7), 67.1 (C-8), 71.4 (C-10), 78.0 (C-9), 113.8 (C-11), 129.6 (C-12), 129.7 (C-13), 159.3 (C-14), 170.7 (C-15); $[\alpha]_D^{24}$ = -36.9 (c=4,7, CH_2Cl_2); IR: 3412, 2952, 2867, 1631, 1512, 1463, 1364, 1300, 1247, 1173, 1081, 1031, 819 cm^{-1}; HRMS (ESI): calcd for $C_{19}H_{30}NO_4$ [M+H]$^+$: 336.2175, found 336.2194.

1-{(2*S*)-2-[(4-Methoxybenzyl)oxy]-4-methylpentanoyl]piperidin-4-one (18)

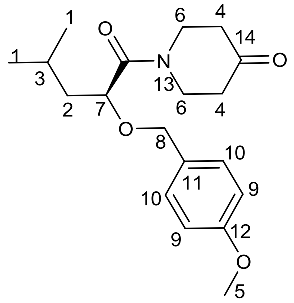

33 mL dry DCM were cooled to -60°C. 810 µL (11.40 mmol) dry DMSO and 1.49 mL (8.56 mmol) oxalylchloride were successively added drop-wise. The mixture was stirred for 30 min and then 1.91 g (5.70 mmol) **17**, dissolved in 3.5 mL dry DCM, were slowly added drop-wise. After 30 min stirring 4.12 mL (28.50 mmol) dry Et_3N were added and the mixture was allowed to warm up to r.t. After 30 min additional stirring the solution was washed twice with aq. tartaric acid (20%, 15 mL, 8 mL) and once with 10 mL sat. $NaHCO_3$. After drying with $MgSO_4$ and evaporating the residue was purified by FC (DCM/MeOH 100:2) to give 1.63 g (4.89 mmol, 86%) **18** as colorless oil. R*f* (DCM/MeOH 100/2) = 0.19; ^{1}H-NMR (300 MHz, $CDCl_3$, ppm): δ 0.88 (d*d, ^3J = 22.8 Hz, ^4J = 6.3 Hz, 6H, H-1), 1,48 (m, 1H, H-2), 1.81 (m, 2H, H-2, H-3), 2.35 (m, 4H, H-4), 3.79 (s, 3H, H-5), 3.92 (m, 4H, H-6), 4.25 (d*d, ^3J = 4.5 Hz, ^4J = 9.6 Hz, 1H, H-7), 4.42 (d*d, ^3J = 41.7 Hz, ^4J = 11.3 Hz 2H, H-8), 6.85 (d, ^3J = 8.7 Hz, 2H, H-9), 7.24 (d, ^3J = 8.7 Hz, 2H, H-10); ^{13}C-NMR (75.5 MHz, $CDCl_3$, ppm): δ 21.7 (C1), 23.1 (C-1), 24.8 (C-3), 41.0 (C-4), 41.3 (C-4), 41.5 (C-2), 41.9 (C-6), 41.4 (C-6), 55.3 (C-5), 71.8 (C-8), 78.9 (C-7), 113.9 (C-9), 129.3 (C-10), 129.6 (C-11), 159.5 (C-12), 171.2 (C-13), 206.8 (C-14); $[\alpha]_D^{22}$ = -30.4 (c=4.7, CH_2Cl_2); IR: 2955,

2868, 1718, 1637, 1512, 1439, 1364, 1302, 1247, 1173, 1084, 1032, 820 cm^{-1}; HRMS (ESI): calcd. for $C_{19}H_{28}NO_4$ [M+H]+: 334.2018, found 336.2019.

_S_-3-(Trimethylsilyl)prop-2-yn-1-yl 15-{2-[(4-methoxybenzyl)oxy]-4-methylpentanoyl}-7,11,18,21-tetraoxa-3,15-diazatrispiro[5.2.2.5^{12}.2^{9}.2^{6}]heneicosan-3-carboxylate (19)

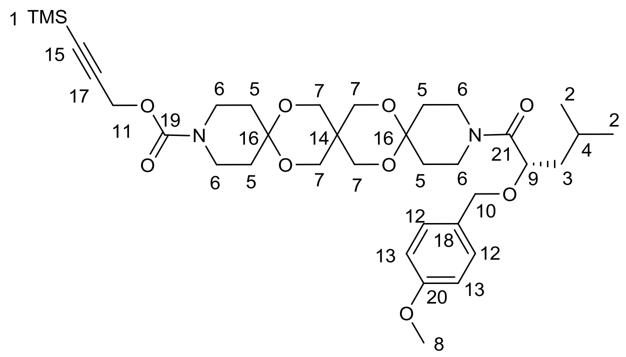

2.24 g (6.73 mmol) of ketone **18** were suspended in 200 mL dry Et_2O and cooled to 0°C. After adding 269 mg (6.73 mmol) NaH (60 % content) and 860 µL (6.73 mmol) TMSCl was mixture was stirred for 30 min at r.t. Then 2.5 g (6.73 mmol) of diol **6** and one drop TMSOTf were added and the mixture was stirred over night. After evaporation the residue was purified by FC ($CHCl_3$/EE 2:1) to give 1.90 g (2.75 mmol, 41%) **19** as white solid. R*f* ($CHCl_3$/EE 2/1) = 0.32; ^{1}H-NMR (300 MHz, $CDCl_3$, ppm): δ 0.17 (s, 9H, H-1), 0.84 (d*d, ^{3}J = 24.3 Hz, ^{4}J = 6.3 Hz, 6H, H-2), 1.39 (m, 1H, H-3), 1.82 (m, 10H, H-3, H-4, H-5), 3.50 (m, 5.5H, H-6), 3,65 (m, 3.5H, H-6), 3,74 (m, 8H, H-7), 3.79 (s, 3H, H-8), 4.19 (d*d, ^{3}J = 9.5 Hz, ^{4}J = 4.0 Hz, 1H, H-9), 4.34 (d*d, ^{3}J = 67.8 Hz, ^{4}J = 11.4 Hz 2H, H-10), 4.687 (s, 2H, H-11), 6.87 (d, ^{3}J = 8.7 Hz, 2H, H-12), 7.23 (d, ^{3}J = 8.4 Hz, 2H, H-13); ^{13}C-NMR (75.5 MHz, $CDCl_3$, ppm): δ -0.3 (C-1), 21.7 (C-2), 23.1 (C-2), 24.7 (C-4), 31.5 (C-5), 33.1 (C-14), 33.6 (C-5), 39.1 (C-6), 40.7 (C-6), 41.2 (C-6), 41.5 (C-3), 53.7 (C-11), 55.2 (C-8), 63.4 (C-7), 71.4 (C-10), 78.3 (C-9), 91.5 (C-15), 96.9 (C-16), 99.8 (C-17), 113.8 (C-12), 129.6 (C-13, C-18), 154.3 (C-19), 159.2 (C-20), 170.7 (C-21); m.p. 57-60 °C; $[\alpha]_D^{24}$ = -19.0 (c=4.5, CH_2Cl_2); IR: 2958, 2868, 1704, 1636, 1512, 1467, 1438, 1362, 1248, 1231, 1095, 1035, 844, 760 cm^{-1}; HRMS (ESI): calcd. for $C_{36}H_{55}N_2O_9Si$ $[M+H]^+$: 687.3677, found 687.3674.

3-(Trimethylsilyl)prop-2-yn-yl 15-[(2*S*)-2-hydroxy-4-methylpentanoyl]-7,11,18,21-tetraoxa-3,15-diazatrispiro[5.2.2.5^{12}.2^{9}.2^{6}]heneicosan-3-carboxylate (20)

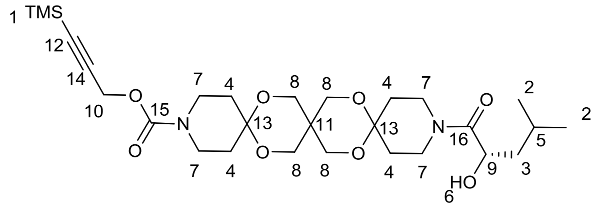

1.81 g (2,63 mmol) **19** and 2.99 g (13,32 mmol) DDQ were dissolved in 528 mL of a mixture of DCM/pH7 buffer (9:1). The dark green mixture was stirred over night and then 176 mL sat. $NaHCO_3$ solution were added. After phase separation the aqueous phase was washed with 117 mL DCM. The combined organic phases were washed with 180 mL sat. $NaHCO_3$ and brine, dried with $MgSO_4$ and evaporated. After purification with FC (DCM/MeOH 100:3) 1.22 g (2.15 mmol, 82%) **20** were obtained as white solid. R*f* (DCM/MeOH 100/3) = 0.24; ^{1}H-NMR (300 MHz, $CDCl_3$, ppm): δ 0.18 (s, 9H, H-1), 0.95 (d*d, ^{3}J = 11.7 Hz, ^{4}J = 6.6 Hz, 6H, H-2), 1.25 (m, 1H, H-3), 1.43 (m, 1H, H-3), 1.85 (m, 8H, H-4), 1.99 (m, 1H, H-5), 2.24 (m, 1H, H-6), 3.36 (m, 2H, H-7), 3.49 (m, 4H, H-7), 3.76 (m, 10H, H-7, H-8), 4.40 (m, 1H, H-9), 4.70 (s, 2H, H-10); ^{13}C-NMR (75.5 MHz, $CDCl_3$, ppm): δ -0.3 (C-1), 21.4 (C-2), 23.6 (C-2), 24.6 (C-5), 31.3 (C-4), 33.1 (C-11), 40.7 (C-7), 44.4 (C-3), 53.8 (C-10), 63.3 (C-8), 66.4 (C-9), 91.6 (C-12), 96.7 (C-13), 99.8 (C-14), 154.3 (C-15), 173.4 (C-16); m.p. 72 °C; $[\alpha]_D^{24}$ = -11.5 (c=3.0, CH_2Cl_2); IR: 3425, 2959, 2875, 1703, 1640, 1468, 1438, 1363, 1249, 1230, 1142, 1095, 1038, 844, 761 cm^{-1}; HRMS (ESI): calcd. for $C_{28}H_{46}N_2O_8NaSi$ $[M+Na]^+$: 589.2911, found 589.2921.

Prop-2-yn-1-yl 15-[(2*S*)-2-hydroxy-4-methylpentanoyl]-7,11,18,21-tetraoxa-3,15-diazatrispiro [5.2.2.5^{12}.2^{9}.2^{6}]heneicosan-3-carboxylate (21)

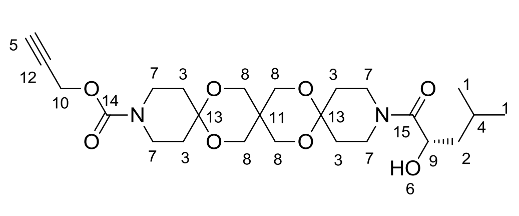

522 mg (921.02 µmol) **20** and 153 mg (1.11 mmol) K_2CO_3 were dissolved in 10 mL MeOH and stirred for 100 min. After addition of 0.01 M HCl the mixture was extracted twice with 15 mL DCM. The combined organic phases were washed with 10 mL brine, dried with $MgSO_4$ and evaporated. The residue was purified by FC (DCM/MeOH 100:3) to give 355 mg (717.38 µmol, 78%) **21** as white solid. R*f* (DCM/MeOH 100/3) = 0.2; ^{1}H-NMR (300 MHz, $CDCl_3$, ppm): δ 0.93 (d*d, ^{3}J = 11.4 Hz, ^{4}J = 6.6 Hz, 6H, H-1), 1.23 (m, 1H, H-2), 1.41 (m, 1H, H-2), 1.84 (m, 8H, H-3), 1.95 (m, 1H, H-4), 2.45 (t, ^{3}J = 2,4 Hz,

1H, H-5), 3.01 (s, 1H, H-6), 3.49 (m, 6H, H-7), 3.74 (m, 10H, H-7, H-8), 4.35 (d*d, ^3J = 10.2 Hz, ^4J = 1.8 Hz, 2H, H-9), 4.67 (d, ^3J = 2.4 Hz, 2H, H-10); ^{13}C-NMR (75.5 MHz, $CDCl_3$, ppm): δ 21.3 (C-1), 23.6 (C-1), 24.6 (C-4), 31.3 (C-3), 33.1 (C-11), 39.2 (C-7), 40.6 (C-7), 40.7 (C-7), 41.3 (C-9), 44.4 (C-4), 52.9 (C-10), 63.3 (C-8), 63.4 (C-8), 66.4 (C-9), 74.4 (C-5), 78.4 (C-12), 96.7 (C-13), 96.9 (C-13), 154.2 (C-14), 173.4 (C-15); m.p. 70-72 °C; $[\alpha]_D^{24}$ = -13.7 (c=4,7, CH_2Cl_2); IR: 3418, 2956, 2869, 1699, 1637, 1515, 1468, 1439, 1363, 1232, 1141, 1096, 1038, 940, 891, 763 cm^{-1}; HRMS (ESI): calcd. for $C_{25}H_{38}N_2O_8Na$ $[M+Na]^+$: 517.2526, found 517.2516.

3-(Trimethylsilyl)prop-2-yn-1-yl 15-[(2S)-2-azido-4-methylpentanoyl]-7,11,18,21-tetraoxa-3,15-diazatrispiro[5.2.2. $5^{12}.2^9.2^6$] heneicosan-3-carboxylate 22

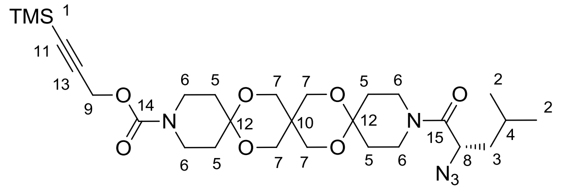

733 mg (1.29 mmol) **20**, 506 mg (1.93 mmol) triphenylphosphane, 126 mg (1.93 mmol) sodium azide and 641 mg (1.93 mmol) tetrabromomethane were dissolved in 30 mL dry DMF and stirred over night. After evaporation and drying in HV the residue was purified by FC (DCM/MeOH 100:2) to give 424 mg (716.50 μmol, 56 %) **22** as white solid. R*f* (DCM/MeOH 100/2) = 0,36; ^{1}H-NMR (300 MHz, $CDCl_3$, ppm): δ 0.17 (s, 9H, H-1), 0.954 (d*d, ^3J = 6.0 Hz, ^4J = 6.0 Hz, 6H, H-2), 1.56 (m, 1H, H-3), 1.82 (m, 10H, H-3, H-4, H-5), 3.496 (m, 8H, H-6), 3.75 (m, 8H, H-7), 3.91 (m, 1H, H-8), 4.69 (s, 2H, H-9); ^{13}C-NMR (75.5 MHz, $CDCl_3$, ppm): δ -0.29 (C-1), 21.8, (C-2), 22.9 (C-2), 25.1 (C-4), 31.2 (C-5), 33.1 (C-10), 33.8 (C-5), 39.1 (C-6) , 39.1 (C-3), 40.7 (C-6), 42.2 (C-6), 53.8 (C-9), 57.0 (C-8), 63.5 (C-7), 91.6 (C-11), 96.7 (C-12), 97.0 (C-12), 99.9 (C-13), 154.3 (C-14), 168.5 (C-15); m.p. 63 °C; $[\alpha]_D^{25}$ = -36.0 (c=3.0, CH_2Cl_2); IR: 2961, 2870, 2107, 1703, 1650, 1468, 1439, 1363, 1249, 1230, 1140, 1094, 1038, 844, 761 cm^{-1}; HRMS (ESI): calcd. for $C_{28}H_{45}N_5O_7NaSi$ $[M+Na]^+$: 614.2986, found 614.2983.

(1-{(2*S*)-4-Methyl-1-oxo-1-[15-({[3-(trimethylsilyl)prop-2-yn-1-yl]oxy}carbonyl)-7,11,18,21-tetraoxa-3,15-diazatrispiro[5.2.2.5^{12}.2^{9}.2^{6}]heneicos-3-yl]pentan-2-yl}-1*H*-1,2,3-triazol-4-yl) methyl-15-[(2*S*)-2-hydroxy-4-methylpentanoyl]-7,11,18,21-tetraoxa-3,15-diazatrispiro[5.2.2. 5^{12}.2^{9}.2^{6}]heneicosan-3-carboxylate (23)

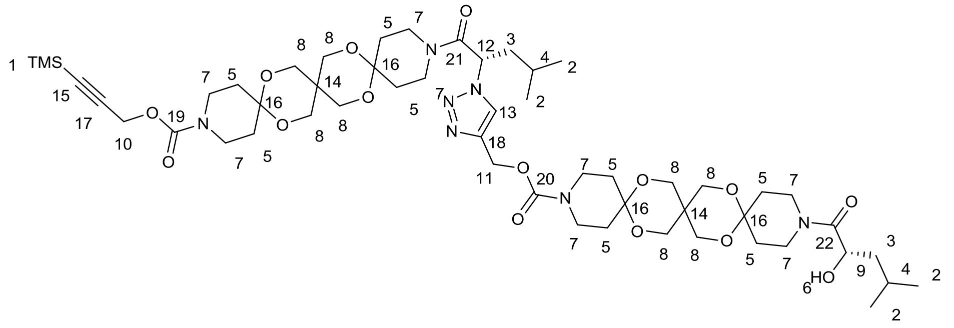

329 mg (665.20 µmol) **21** and 398 mg (671.87 µmol) **22** dissolved in 1.33 mL DCM. 95 µL (686.63 µmol) Et_3N and 33 mg Cu/C catalyst (1.01 mmol/g) were added and the mixture was stirred over night. After evaporation the residue was purified by FC (DCM/MeOH 100:4) to give 533 mg (490.61 µmol, 74%) **23** as white solid. R*f* (DCM/MeOH 100:4) = 0.26; ^{1}H-NMR (300 MHz, $CDCl_3$, ppm): δ 0.15 (s, 9H, H-1), 0.93 (m, 12H, H-2), 1.22 (m, 3H, H-3), 1.59 (m, 1H, H-3), 1.81 (m, 18H, H-4, H-5), 2.82 (m, 1H, H-6), 3.47 (m, 12H, H-7), 3.72 (m, 20H, H-7, H-8), 4.37 (m, 1H, H-9), 4.66 (s, 2H, H-10), 5.17 (s, 2H, H-11), 5.79 (t, ^3J = 7.5 Hz, 1H, H-12), 7.86 (s, 1H, H-13); ^{13}C-NMR (75.5 MHz, $CDCl_3$, ppm): δ -0.3 (C-1), 21.3 (C-2), 21.7 (C-2), 22.5 (C-2), 23.6 (C-2), 24.5 (C-4), 24.6 (C-4), 31.3 (C-5), 31.6 (C-5), 33.0 (C-14), 39.3 (C-7), 40.6 (C-7), 41.3 (C-7), 41.8 (C-3), 42.6 (C-7), 44.4 (C-3), 53.7 (C-10), 57.7 (C-12), 58.4 (C-11), 63.2 (C-8), 66.3 (C-9), 91.5 (C-15), 96.9 (C-16), 99.8 (C-17), 122.6 (C-13), 143.6 (C-18), 154.2 (C-19), 154.8 (C-20), 166.1 (C-21), 173.3 (C-22); m.p. 111-115 °C; $[\alpha]_D^{27}$ = -27.6 (c=4.2, CH_2Cl_2); IR: 3448, 2960, 2870, 2105, 1699, 1653, 1469, 1439, 1363, 1231, 1166, 1141, 1094, 1039, 940, 892, 845, 762 cm^{-1}; HRMS (ESI): calcd. for $C_{53}H_{84}N_7O_{15}Si$ $[M+H]^+$: 1086.5795, found 1086.5739.

{1-[2*S*)-4-Methyl-1-oxo-1-{15-[prop-2-yn-1-yloxy)carbonyl]-7,11,18,21-tetraoxa-3,15-diazatrispiro[5.2.2.5^{12}.2^{9}.2^{6}]heneicos-3-yl}pentan-2-yl]-1*H*-1,2,3-triazol-4-yl}methyl-15-[(2*S*)-2-hydroxy-4-methylpentanoyl]-7,11,18,21-tetraoxa-3,15-diazatrispiro[5.2.2.5^{12}.2^{9}.2^{6}]heneicosan-3-carboxylate (24)

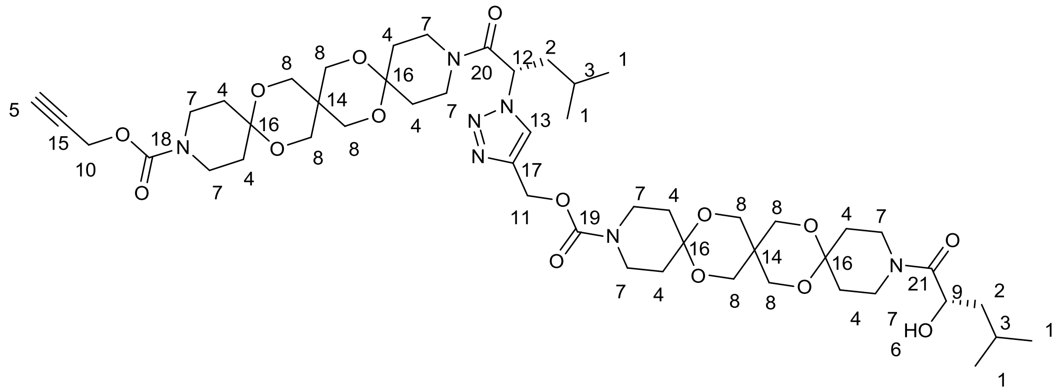

520 mg (478.67 µmol) of compound **23** and 79 mg (574.40 µmol) K_2CO_3 were dissolved in 7 mL MeOH and stirred for 60 min. After addition of 2 ml 0.01 M HCl the solution was extracted twice with 15 mL DCM. The combined organic phases were washed with 10 mL brine, dried with $MgSO_4$ and evaporated. 390 mg (384.54 µmol, 79%) **24** were obtained as white solid after FC (DCM/MeOH 100:4). R*f* (DCM/MeOH 100:4) = 0.25; ^{1}H-NMR (300 MHz, $CDCl_3$, ppm): δ 0.91 (m, 12H, H-1), 1.22 (m, 3H, H-2, H-3), 1.57 (m, 1H, H-2, H-3), 1.81 (m, 18H, H-2, H-3, H-4), 2.44 (t, ^3J = 2.4 Hz, 1H, H-5), 2.96 (s, 1H, H-6), 3.46 (m, 14H, H-7), 3.71 (m, 20H, H-7, H-8), 4.36 (m, 1H, H-9), 4.65 (d, ^3J = 2.4 Hz, 2H, H-10), 5.17 (s, 2H, H-11), 5.78 (t, ^3J = 7.5 Hz, 1H, H-12), 7.86 (s, 1H, H-13); ^{13}C-NMR (75.5 MHz, $CDCl_3$, ppm): δ 21.3 (C-1), 21.7 (C-1), 22.5 (C-1), 23.5 (C-1), 24.5 (C-2), 31.3 (C-4), 31.6 (C-4), 33.0 (C-14), 39.3 (C-7), 40.6 (C-7), 41.3 (C-7), 41.8 (C-3), 42.5 (C-7), 44.3 (C-3), 52.9 (C-10), 57.7 (C-12), 58.4 (C-11), 63.4 (C-8), 66.3 (C-9), 74.4 (C-5), 78.4 (C-15), 96.6 (C-16), 122.6 (C-13), 143.6 (C-17), 154.2 (C-18), 154.7 (C-19), 166.1 (C-20), 173.3 (C-21); m.p. 106-108 °C; $[\alpha]_D^{27}$ = -24.9 (c=2.8, CH_2Cl_2); IR: 3457, 2960, 2870, 1703, 1642, 1469, 1439, 1363, 1273, 1232, 1166, 1142, 1094, 1041, 940, 892, 847, 796, 763, 730, 651 cm^{-1}; HRMS (ESI): calcd. for $C_{50}H_{76}N_7O_{15}$ $[M+H]^+$: 1014.5399, found 1014.5380.

[1-((2*S*)-4-Methyl-1-oxo-1-{15-[(prop-2-yn-1-yloxy)carbonyl]-7,11,18,21-tetraoxa-3,15-diazatrispiro[5.2.2.5^{12}.2^{9}.2^{6}]heneicos-3-yl}pentan-2-yl)-1*H*-1,2,3-triazol-4-yl]methyl-15-[(2*S*)-2-azido-4-methylpentanoyl]-7,11,18,21-tetraoxa-3,15-diazatrispiro[5.2.2. 5^{12}.2^{9}.2^{6}]heneicosan-3-carboxylate (25)

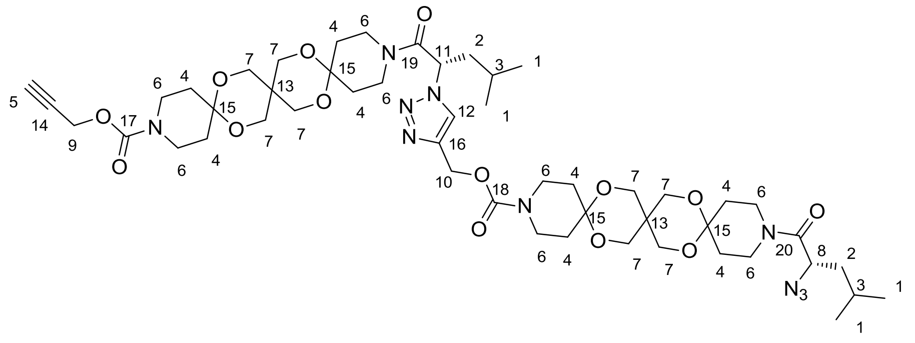

350 mg (345.11 µmol) **24**, 136 mg (517 µmol) triphenylphosphane, 67.3 mg (517 µmol) sodium azide and 172 mg (517 µmol) tetrabromomethane were dissolved in 9 mL dry DMF and stirred for 2h. After evaporation and drying in HV the residue was purified by FC (DCM/MeOH 100:4) to give 270 mg (260 µmol, 75%) **25** as white solid. R*f* (DCM/MeOH 100:5) = 0.38; ^{1}H-NMR (300 MHz, $CDCl_3$, ppm): δ 0.92 (m, 12H, H-1), 1.30 (m, 1.5H, H-2, H-3), 1.56 (m, 2.5H, H-2, H-3), 1.79 (m, 18H, H-2, H-3, H-4), 2,44 (t, ^{3}J = 2.25 Hz, 1H, H-5), 3.46 (m, 13H, H-6), 3.71 (m, 19H, H-6, H-7), 3.89 (m, 1H, H-8), 4.65 (d, ^{3}J = 2.1 Hz, 2H, H-9), 5.17 (s, 2H, H-10), 5.78 (t, ^{3}J = 6.6 Hz, 1H, H-11), 7.86 (s, 1H, H-12); ^{13}C-NMR (75.5 MHz, $CDCl_3$, ppm): δ 21.7 (C-1), 22.5 (C-1), 22.8 (C-1), 24.5 (C-2), 25.0 (C-2), 31.2 (C-4), 31.6 (C-4), 33.0 (C-13), 33.6 (C-4), 39.3 (C-6), 39.4 (C-6), 40.6 (C-6), 41.7 (C-3), 42.5 (C-3), 52.9 (C-9), 56.9 (C-8), 57.6 (C-11), 58.4 (C-10), 63.2 (C-7), 74.4 (C-5), 78.4 (C-14), 96.8 (C-15), 122.6 (C-12), 143.6 (C-16), 154.1 (C-17), 154.7 (C-18), 166.1 (C-19), 168.4 (C-20); m.p. 88-90 °C; $[\alpha]_D^{25}$ = -50.5 (c=3.5, CH_2Cl_2); IR: 2961, 2870, 2104, 1699, 1655, 1468, 1440, 1363, 1272, 1231, 1198, 1140, 1093, 1042, 940, 892, 795, 762, 729 cm^{-1}; HRMS (ESI): calcd. for $C_{50}H_{75}N_{10}O_{14}$ $[M+H]^{+}$: 1039.5459, found 1039.5457.

(12*S*,34*S*)-12,34-Bis(2-methylpropyl)-2,6,18,24,28,40,50,53,59,62-decaoxa-10,13,14,15,20,32,35,36,37,42-decaazaundecacyclo[40.2.2.2^{1,4}.2^{4,7}.2^{7,10}.2^{20,23}.2^{23,26}.2^{26,29}.2^{29,32}.1^{13,16}.1^{35,38}]dohexaconta-14,16(56),36,38(47)-tetraen-11,19,33,41-tetron (26)

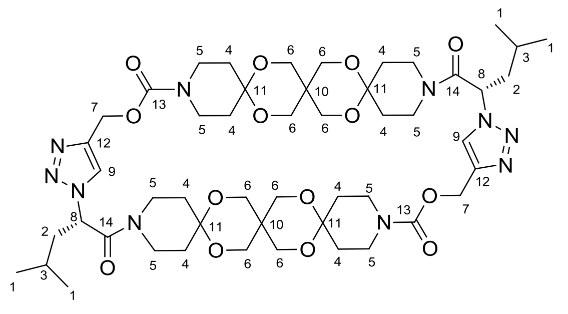

8.8 µL (57,7 µmol) DBU and 8 mg (57,7 µmol) CuBr were dissolved in 24 mL dry DCM. The solution was protected against light and a solution of 61 mg (57 µmol) **25**, dissolved in 2 mL DCM, were added slowly using a syringe driver (flow 1mL/h). After complete addition the mixture was stirred over night, filtered and washed successively with 10 mL 0,01 M HCl, sat. $NaHCO_3$ and brine. After drying and evaporating the residue was purified by FC (DCM/MeOH 10:1) to give 18 mg (17.42 µmol; 30%) **26** as white solid. R*f* (DCM/MeOH 100/5) = 0.34; ^{1}H-NMR (300 MHz, $CDCl_3$, ppm): δ 1.00 (d*d, ^3J = 31.1 Hz, ^4J = 6.5 Hz, 12H, H-1), 1.26 (m, 4H, H-2, H-3), 1,56 (m, 2H, H-2, H-3), 1.92 (m, 16H, H-4), 2.78 (m, 1.5H, H-5), 3.10 (m, 2H, H-5), 3.27 (m, 4H, H-5), 3.56 (m, 23H, H-5, H-6), 4.41 (m, 1,5H, H-5), 5.11 (m, 4H, H-7), 5.67 (m, 1H, H-8), 7.65 (s, 2H, H-9); ^{13}C-NMR (75.5 MHz, $CDCl_3$, ppm): δ 22.3 (C-1), 23.1 (C-1), 24.7 (C-2), 30.1 (C-4), 32.6 (C-4), 33.4 (C-10), 40.1 (C-5), 40.8 (C-5), 42.8 (C-3), 42.8 (C-5), 58.6 (C-7), 58.9 (C-8), 63.5 (C-6), 97.3 (C-11), 122.1 (C-9), 144.2 (C-12), 154.9 (C-13), 165.4 (C-14); $[\alpha]_D^{23}$ = 31.7 (c=2.9, CH_2Cl_2); IR: 2959, 2869, 1694, 1649, 1437, 1365, 1233, 1141, 1093, 1040, 943, 896, 801, 751, 662 cm^{-1}; HRMS (ESI): calcd. for $C_{50}H_{75}N_{10}O_{14}$ $[M+H]^+$: 1039.5459, found 1039.5452.

1-(4-Hydroxypiperidin-1-yl)-2-(pyren-1-yl)ethanone (pre-27a)

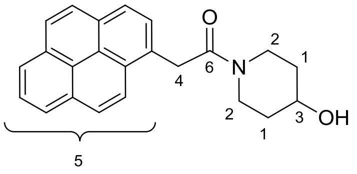

1,42 g (5.44 mmol) Pyrene-1-yl acetic acid, 500 mg (4.94 mmol) 4-hydroxypiperidine and 735 mg (5.44 mmol) HOBT were dissolved in 11 mL dry THF. After 30min stirring 1.12 g (5.44 mmol) DCC were added and the mixture was stirred over night. The precipitate (dicyclohexyl urea) was filtered

off and washed with EE. The combined organic solutions were evaporated, dissolved in 13 mL EE and washed successively with 10 mL 0,01M HCl, 10 mL sat. $NaHCO_3$ and 10 mL brine. After drying with $MgSO_4$ and evaporating the residue was purified by FC (DCM/MeOH 100:3) 1.16 g (3.37 mmol, 68%) **pre-27a**. R*f* (DCM/MeOH 100/3) = 0.12; ^{1}H-NMR (300 MHz, $CDCl_3$, ppm): δ 1.12 (m, 1H, H-1), 1.39 (m, 2H, H-1), 1.69 (m, 2H, H-1), 2.97 (m, 1H, H-2), 3.11 (m, 1H, H-2), 3.60 (m, 2H, H-2, H-3), 4.03 (m, 1H, H-2), 4.28 (s, 2H, H-4), 7.79 (m, 1H, H-5), 7.99 (m, 3H, H-5), 8.09 (m, 5H, H-5); ^{13}C-NMR (75,5 MHz, $CDCl_3$, ppm): δ 33.8 (C-1), 38.6 (C-4), 39.2 (C-2), 43.1 (C-2), 66.5 (C-3), 122.8 (C_{ar}H), 124.6 (C_{ar}), 124.8 (C_{ar}H), 124.9 (C_{ar}), 125.0 (C_{ar}H), 125.2 (C_{ar}H), 125.9 (C_{ar}H), 126.8 (C_{ar}H), 127.1 (C_{ar}H), 127.3 (C_{ar}H), 127.8 (C_{ar}H), 129.0 (C_{ar}), 129.0 (C_{ar}), 130.4 (C_{ar}), 130.7 (C_{ar}), 131.2 (C_{ar}), 169.5 (C-6); m.p. 68-72 °C; IR: 3399, 3040, 2924, 2855, 1619, 1447, 1362, 1329, 1263, 1210, 1181, 1113, 1073, 1022, 977, 930, 845, 756, 710 cm^{-1}; HRMS (EI): calcd. for $C_{23}H_{21}O_2N$ $[M]^+$: 343.1572, found 343.1570.

1-(Pyren-1-ylacetyl)piperidin-4-one (27a)

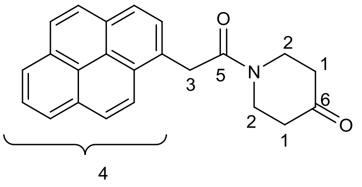

824 mg (2.4 mmol) **pre-27a** were dissolved in 50 mL dry DCM. The solution was cooled with an ice bath and 137 mg (3.1 mmol) DMP were added slowly. After 30min a white precipitate is formed. After additional stirring at r.t. the mixture was washed 7 times with DM washing solution and once with brine. The solution was dried with $MgSO_4$, evaporated and the residue was purified by FC (DCM/MeOH 100:2) to give 726 mg (2.1 mmol, 89%) **27a** as white solid. R*f* (DCM/MeOH 100:2) = 0.18; ^{1}H-NMR (300 MHz, $CDCl_3$, ppm): δ 2.02 (t, ^3J = 5.9 Hz, 2H, H-1), 2.41 (t, ^3J = 6 Hz, 2H, H-1), 3.63 (t, 2H, ^3J = 6 Hz, H-2), 3.92 (t, 2H, ^3J = 6 Hz, H-2), 4.39 (s, 2H, H-3), 7.84 (m, 1H, H-4), 7.86 (m, 3H, H-4), 8.12 (m, 2H, H-5), 8.17 (m, 3H, H-4); ^{13}C-NMR (75.5 MHz, $CDCl_3$, ppm): δ 38.9 (C-3), 40.7 (C-1), 41.1 (C-2), 44.4 (C-2), 122.6 (C_{ar}H), 124.6 (C_{ar}), 124.8 (C_{ar}H), 125.0 (C_{ar}), 125.1 (C_{ar}H), 125.3 (C_{ar}H), 126.0 (C_{ar}H), 126.7 (C_{ar}H), 127.2 (C_{ar}H), 127.3 (C_{ar}H), 128.1 (C_{ar}H), 128.3 (C_{ar}), 129.0 (C_{ar}), 130.5 (C_{ar}), 130.6 (C_{ar}), 131.2 (C_{ar}), 169.9 (C-5), 206.3 (C-6); Fp = 171-173 °C; IR: 3037, 2955, 1714, 1651, 1438, 1375, 1315, 1270, 1222, 1194, 1085, 980, 849, 834, 749, 710 cm^{-1}; HRMS (ESI): calcd. for $C_{23}H_{20}O_2N$ $[M+H]^+$: 342.1489, found 342.1491.

(2*E*)-1-(4-Hydroxypiperidin-1-yl)-3-phenylprop-2-en-1-one (pre-27b)

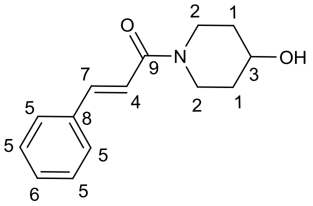

1 g (9,89 mmol) 4-Hydroxypiperidine and 1.73 mL (10,2 mmol) DIEA were dissolved in 20 mL DCM and cooled to 0°C. 1.42 mL (9,89 mmol) cinnamoyl chloride were added drop-wise and the mixture was stirred at r.t. over night. 10 mL 0.02 M HCL were added, the phases were separated and the organic phase was dried with $MgSO_4$ and evaporated. The residue was purified by FC (DCM/MeOH 100:3) to give 2.15 g (9.30 mmol, 94%) **pre-27b** as white solid. R*f* (DCM/MeOH 100/4) = 0.19; ^{1}H-NMR (300 MHz, $CDCl_3$, ppm): δ 1.59 (m, 2H, H-1), 1.92 (m, 2H, H-1), 3.37 (m, 2H, H-2), 4.01 (m, 3H, H-2, H-3), 6,92 (d, ^{3}J = 15.3 Hz, 1H, H-4), 7.35 (m, 3H, H-5, H-6), 7.50 (m, 2H, H-5), 7.67 (d, ^{3}J = 15,3 Hz, 1H, H-7) ; ^{13}C-NMR (75,5 MHz, $CDCl_3$, ppm): δ 33.9 (C-1), 33.5 (C-2), 43.0 (C-2), 66.4 (C-3), 117.2 (C-5), 127.5 (C-5), 128.6 (C-5), 129.4 (C-6), 135.0 (C-8), 142.4 (C-7), 165.4 (C-9); IR: 3399, 2941, 2867, 1640, 1585, 1495, 1454, 1438, 1363, 1279, 1254, 1216, 1113, 1084, 1021, 972, 758, 701 cm^{-1}; HRMS (EI): calcd. for $C_{14}H_{17}O_2N$ $[M]^+$: 231.1259, found 231.1254.

1-[(2*E*)-3-Phenylprop-2-enoyl]piperidin-4-one (27b)

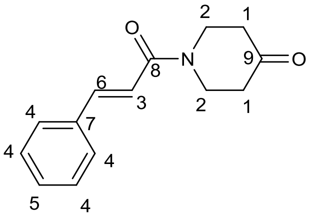

1,6 g (6.90 mmol) **pre-27b** were dissolved in 140 mL dry DCM. After cooling to 0°C 3,67 g (8,65 mmol) DMP were added slowly. The mixture was stirred for 90 min at r.t. and afterwards extracted 7 times with DM washing solution (200 mL in each case). The organic phase was dried with $MgSO_4$ and evaporated. After FC (DCM/MeOH 100:2) 1.37 g (5.97 mmol, 86%) **27b** were obtained as white solid. R*f* (DCM/MeOH 100/2) = 0.18; ^{1}H-NMR (300 MHz, $CDCl_3$, ppm): δ 2.52 (m, 4H, H-1), 3.95 (m, 4H, H-2) 6.95 (d, ^{3}J = 15.3 Hz, 1H, H-3), 7.36 (m, 3H, H-4, H-5), 7.53 (m, 2H, H-4), 7.75 (d, ^{3}J = 15.6 Hz, 1H, H-7) ; ^{13}C-NMR (75.5 MHz, $CDCl_3$, ppm): δ 41.0 (C-1), 44.2 (C-2), 116.3 (C-3), 127.8 (C-4), 128.8 (C-4), 129.8 (C-5), 134.9 (C-7), 143.7 (C-6), 165.7 (C-8), 206.5 (C-10); m.p. 106-108 °C; IR: 2964, 1710, 1640, 1599,

1458, 1438, 1357, 1273, 1258, 1229, 1192, 1093, 977, 765, 682 cm^{-1}; HRMS (ESI): calcd. for $C_{14}H_{16}O_2N$ $[M+H]^+$: 230.1176, found 230.1181.

Prop-2-yn-1-yl 3,3-bis(hydroxymethyl)-1,5-dioxa-9-azaspiro[5.5]undecan-9-carboxylate (28)

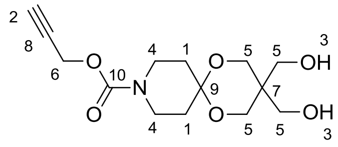

3 g (8.08 mmol) of compound **6** and 1.34 g (9,69 mmol) K_2CO_3 were dissolved in 100 mL MeOH and stirred for 3h. After evaporation 40mL 0.01 M HCl were added and the mixture was washed twice with 200 mL DCM. The combined organic phases were washed with brine, dried with $MgSO_4$ and evaporated. After purification with FC (DCM/MeOH 100:5) 2,28 g (7.44 mmol, 92%) **28** were obtained as white solid. R*f* (DCM/MeOH 100/5) = 0,22 ; ^{1}H-NMR (300 MHz, $CDCl_3$, ppm): δ 1.81 (m, 4H, H-1), 2.46 (s, 1H, H-2), 2.76 (s, 2H, H-3), 3.48 (m, 4H, H-4), 3.71 (s, 8H, H-5), 4.67 (s, 2H, H-6); ^{13}C-NMR (75.5 MHz, $CDCl_3$, ppm): δ 32.8 (C-1), 32.8 (C-1), 33.5 (H-4), 39.7 (C-7) , 41.2 (C-4), 53.4 (C-6), 62.5 (C-5), 63.8 (C-5), 64.9 (C-5), 75.0 (C-2), 78.8 (C-8), 97.0 (C-9), 154.73 (C-10); m.p. 106,9°C; IR: 3276, 2962, 2883, 2128, 1706, 1468, 1417, 1356, 1269, 1225, 1137, 1056, 999, 952, 937, 899, 880, 766, 71, 665 cm^{-1}; HRMS (ESI): calcd. for $C_{14}H_{21}NNaO_6$ $[M+Na]^+$: 322.1261, found 322.1249.

2-Azido-1-[3,3-bis(hydroxymethyl)-1,5-dioxa-9-azaspiro[5.5]undec-9-yl]ethanone (29)

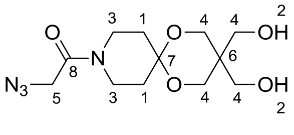

2.85 g (9,72 mmol) 1-[3,3-Bis(hydroxymethyl)-1,5-dioxa-9-azaspiro[5.5]undec-9-yl]-2-chloroethanone **27c** [3] were dissolved in 30 mL dry DMF and 758 mg (11.66 mmol) NaN_3 were added. The mixture was stirred for 2h at r.t., evaporated and dried in HV. The residue was purified by FC (DCM/MeOH 100:6) to give 2.80 g (9.31 mmol, 96%) **29** as white solid. R*f* (DCM/MeOH 100:8) = 0.33; ^{1}H-NMR (300 MHz, CD_3OD, ppm): δ 1.88 (m, 4H, H-1), 3.30 (m, 1H, H-2), 3.40 (m, 2H, H-3), 3.58 (m, 6H, H-3, H-4), 3.76 (s, 4H, H-4), 4.10 (s, 2H, H-5); ^{13}C-NMR (75.5 MHz, CD_3OD, ppm): δ 33.1 (C-1), 34.0 (C-1), 40.3 (C-6), 40.8 (C-3), 43.0 (C-3), 62.7 (C-4), 97.4 (C-7), 168.1 (C-8).

Prop-2-yn-1-yl 15-(pyren-1-ylacetyl)-7,11,18,21-tetraoxa-3,15-diazatrispiro [5.2.2.5^{12}.2^{9}.2^{6}]heneicosan-3-carboxylate (30a)

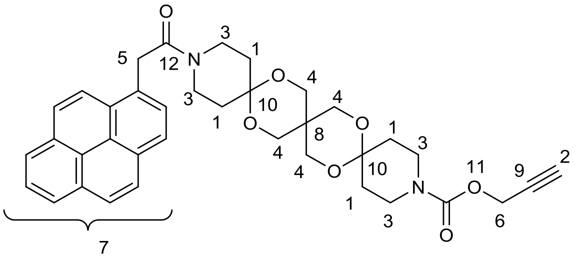

227 mg (666.86 µmol) of ketone **27a** were suspended in 20 mL dry Et_2O and cooled to 0°C. 27 mg (675.00 µmol) NaH (60% content) and 85 µL (675.00 µmol) TMSCl were added and the mixture was stirred 1h at r.t. After this, 200 mg (668.23 µmol) of diol **28** and a drop TMSOTf and the mixture was stirred over night. After evaporation the residue was purified by FC (DCM/MeOH 100:3) to give 192 mg (308.33 µmol, 46%) **30a** as white solid. R*f* (DCM/MeOH 100/3) = 0,27; ^{1}H-NMR (300 MHz, $CDCl_3$, ppm): δ 1.47 (m, 2H, H-1), 1.76 (m, 6H, H-1), 2.47 (t, ^3J = 2.6 Hz, 1H, H-2), 3.46 (m, 7H, H-3), 3.61 (m, 9H, H-3, H-4), 4.35 (s, 2H, H-5), 4.69 (d, ^3J = 2.4 Hz, 2H, H-6); ^{13}C-NMR (75.5 MHz, $CDCl_3$, ppm): δ 31.4 (C-1), 32.9 (C-8), 32.9 (C-1), 38.7 (C-5), 40.6 (C-3), 42.6 (C-3), 52.8 (C-6), 63.2 (C-4), 74.4 (C-2), 78.4 (C-9), 96.7 (C-10), 122.7 (C_{ar}H), 124.6 (C_{ar}), 124.8 (C_{ar}H), 124.9 (C_{ar}), 125.0 (C_{ar}H), 125.1 (C_{ar}H), 125.9 (C_{ar}H), 126.6 (C_{ar}H), 127.0 (C_{ar}H), 127.3 (C_{ar}H), 127.8 (C_{ar}H), 128.9 (2C_{ar}), 130.3 (C_{ar}), 130.6 (C_{ar}), 131.2 (C_{ar}), 154.1 (C-11), 169.3 (C-12); m.p. 115-116 °C; IR: 3249, 2934, 2868, 1702, 1640, 1438, 1361, 1230, 1093, 940, 890, 846, 760, 711 cm^{-1}; HRMS (ESI): calcd. for $C_{37}H_{39}N_2O_7$ $[M+H]^+$: 623.2752, found 623.2753.

Prop-2-in-1-yl 15-[(2*E*)-3-phenylprop-2-enoyl]-7,11,18,21-tetraoxa-3,15-diazatrispiro [5.2.2.5^{12}.2^{9}.2^{6}]heneicosan-3-carboxylate (30b)

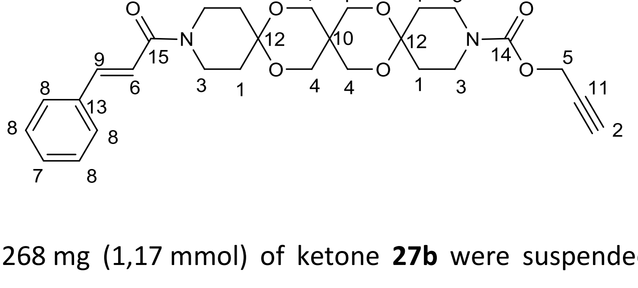

268 mg (1,17 mmol) of ketone **27b** were suspended in 35 mL dry Et_2O and cooled to 0 °C. After addition of 47 mg (1.17 mmol) NaH (60% content) and 150 µL (1.17 mmol) TMSCl the mixture was stirred for 1h at r.t.. After this, 350 mg (1.17 mmol) of diol **28** and a drop TMSOTf were added and the mixture was stirred over night. After evaporation the residue was purified by FC (DCM/MeOH

100:2) to give 267 mg (522.91 µmol, 45%) **30b** as white solid. R*f* (DCM/MeOH 100/3) = 0.27; ^{1}H-NMR (300 MHz, $CDCl_3$, ppm): δ 1.84 (m, 8H, H-1), 2.45 (t, ^{3}J=2.4 Hz, 1H, H-2), 3.46 (m, 4H, H-3), 3.72 (m, 12H, H-3, H-4), 4.64 (d, 2.4 Hz, 2H, H-5), 6.88 (d, ^{3}J = 15.6 Hz, 1H, H-6), 7.31 (m, 3H, H-7, H-8), 7.5 (m, 2H, H-8), 7.6 (d, ^{3}J = 15.6 Hz, 1H, H-9); ^{13}C-NMR (75.5 MHz, $CDCl_3$, ppm): δ 31.3 (C-1), 32.6 (C-1), 33.0 (C-10), 40.6 (C-3), 52.8 (C-5), 63.3 (C-4), 74.4 (C-2), 78.4 (C-11), 96.9 (C-12), 117.1 (C-6), 127.6 (C-8), 128.6 (C-8), 129.4 (C-7), 135.1 (C-13), 142.5 (C-9), 154.1 (C-14), 165.3 (C-15); m.p. 83.8-85.0 °C; IR: 3246, 2937, 2869, 1699, 1645, 1604, 1438, 1362, 1231, 1199, 1165, 1094, 1037, 976, 940, 894, 762, 705 cm^{-1}; HRMS (ESI): calcd. for $C_{28}H_{35}N_2O_7$ $[M+H]^+$: 511.2439, found 511.2441.

Prop-2-yn-1-yl 15-(chloroacetyl)-7,11,18,21-tetraoxa-3,15-diazatrispiro[5.2.2.5^{12}.2^{9}.2^{6}]heneicosan-3-carboxylate (30c)

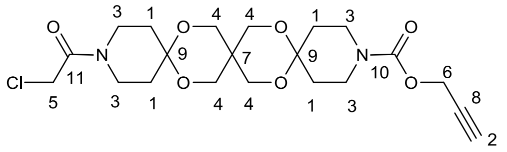

763 mg (4.35 mmol) 1-[3,3-Bis(hydroxymethyl)-1,5-dioxa-9-azaspiro[5.5]undec-9-yl]-2-chloro-ethanone **27c** [3] were suspended in 130 mL dry Et_2O and cooled to 0°C. 174 mg (4.35 mmol) NaH (60% content) und 555 µL (4.35 mmol) TMSCl were added and the mixture was stirred 45 min at r.t.. After this, 1.3 g (4.34 mmol) of diol **28** and a drop TMSOTf were added and the mixture was stirred over night. On the next day the mixture was evaporated and the residue was purified by FC (DCM/MeOH 100:2) to give 1.20 g (2.63 mmol, 60%) **30c** as white solid. R*f* (DCM/MeOH 100/3) = 0.26; ^{1}H-NMR (300 MHz, $CDCl_3$, ppm): δ 1.79 (m, 8H, H-1), 2.44 (t, ^{3}J = 2.1 Hz, 1H, H-2), 3.47 (m, 6H, H-3), 3.58 (m, 2H, H-3), 3.72 (m, 8H, H-4), 4.04 (s, 2H, H-5), 4.65 (d, ^{3}J = 2.1 Hz, 2H, H-6); ^{13}C-NMR (75.5 MHz, $CDCl_3$, ppm): δ 31.07 (C-1), 33.01 (C-7), 33.25 (C-1), 38.9 (C-3), 40.7 (C-3), 40.9 (C-5), 43.0 (C-3), 52.8 (C-6), 63.2 (C-4), 74.4 (C-2), 78.4 (C-8), 96.8 (C-9), 154.1 (C-10), 164.8 (C-11); m.p. 53-55 °C; IR: 3289, 2966, 2869, 1700, 1649, 1469, 1439, 1363, 1275, 1231, 1199, 1166, 1143, 1094, 1036, 940, 888, 788 762, 730, 656 cm^{-1}; HRMS (ESI): calcd. for $C_{21}H_{30}ClN_2O_7$ $[M+H]^+$: 457.1736, found 457.1744.

2-Azido-1-[15-(pyren-1-ylacetyl)-7,11,18,21-tetraoxa-3,15-diazatrispiro[5.2.2.5^{12}.2^{9}.2^{6}]heneicos-3-yl]ethanone (31a)

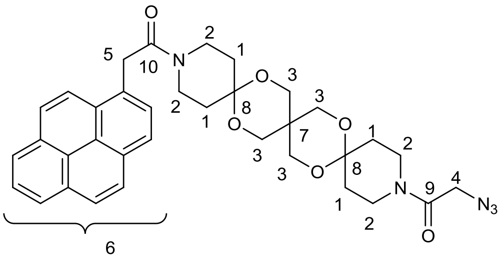

231 mg (675.68 µmol) of ketone **27a** were suspended in 20 mL dry Et_2O and cooled to 0 °C. 27 mg (675.00 µmol) NaH (60% content) and 85 µL (675.00 µmol) TMSCl were added and the mixture was stirred 1h. After this, 200 mg (665.98 µmol) of diol **29** and a drop TMSOTf were added and stirring was continued over night. On the next day the mixture was evaporated and the residue was purified by FC (DCM/MeOH 100:2) to give 258 mg (413.66 µmol, 62%) **31a** as white solid. R*f* (DCM/MeOH 100:3) = 0,21; ^{1}H-NMR (300 MHz, $CDCl_3$, ppm): δ 1.49 (m, 2H, H-1), 1.79 (m, 6H, H-1), 3.19 (m, 2H, H-2), 3.39 (m, 2H, H-2), 3.59 (m, 12H, H-2, H-3), 3.83 (s, 2H, H-4) 4.35 (s, 2H, H-5); ^{13}C-NMR (75.5 MHz, $CDCl_3$, ppm): δ 31.0 (C-1), 31.4 (C-1), 31.4 (C-1), 32.9 (C-7), 33.1 (C-2), 38.6 (C-5), 38.7 (C-2), 41.5 (C-2), 42.6 (C-2), 50.5 (C-4), 63.1 (C-3), 96.4 (C-8), 96.7 (C-8), 122.7 (C_{ar}H), 124.6 (C_{ar}), 124.8 (C_{ar}H), 124.9 (C_{ar}), 125.0 (C_{ar}H), 125.1 (C_{ar}H), 125.9 (C_{ar}H), 126.7 (C_{ar}H), 127.0 (C_{ar}H), 127.2 (C_{ar}H), 127.8 (C_{ar}H), 128.9 (C_{ar}), 128.9 (C_{ar}), 130.3 (C_{ar}), 130.6 (C_{ar}), 131.2 (C_{ar}), 165.2 (C-9), 169,3 (C-10); m.p. 110 °C; IR: 2867, 2104, 1645, 1440, 1362, 1245, 1221, 1165, 1144, 1090, 1036, 940, 908, 888, 847, 796, 711 cm^{-1}; HRMS (ESI): calcd. for $C_{35}H_{38}N_5O_6$ $[M+H]^+$: 624.2817, found 624.2815.

(2*E*)-1-[15-Azidoacetyl)-7,11,18,21-tetraoxa-3,15-diazatrispiro[5.2.2.5^{12}.2^{9}.2^{6}]heneicos-3-yl]-3-phenylprop-2-en-1-one (31b)

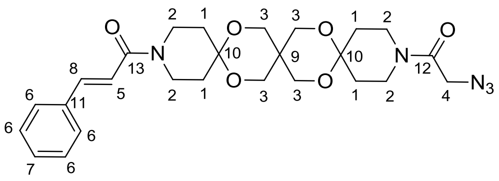

191 mg (832.97 µmol) of ketone **27b** were suspended in 25 mL dry Et_2O and cooled to 0 °C. 33 mg (832.97 µmol) NaH (60% content) and 106 µL (832.97 µmol) TMSCl were added and the mixture was stirred for 1h. After this, 250 mg (832.50 µmol) of diol **29** and a drop TMSOTf were added and stirring was continued over night. On the next day, the mixture was evaporated and the residue was purified

by FC (DCM/MeOH 100:2) to give 250 mg (488.69 µmol, 59%) **31b** as white solid. R*f* (DCM/MeOH 100/3) = 0.16; ^{1}H-NMR (300 MHz, $CDCl_3$, ppm): δ 1.83 (m, 8H, H-1), 3.31 (m, 2H, H-2), 3.68 (m, 14H, H-2, H-3), 3.89 (s, 2H, H-4), 6.88 (d, ^{3}J = 15.3 Hz, 1H, H-5), 7.31 (m, 3H, H-6, H-7), 7.46 (m, 2H, H-6), 7.62 (d, ^{3}J = 15.3 Hz, 1H, H-8) ; ^{13}C-NMR (75.5 MHz, $CDCl_3$, ppm): δ 31.0 (C-1), 33.0 (C-9), 33.3 (C-1), 38.8 (C-2), 41.6 (C-2), 50.5 (C-4), 63.2 (C-3), 96.5 (C-10), 96.9 (C-10), 117.1 (C-5), 127.5 (C-6), 128.6 (C-6), 129.4 (C-7), 135.1 (C-11), 142.5 (C-8), 165.2 (C-12), 165.3 (C-13); m.p. 80.4-82.8 °C; IR: 2934, 2869, 2105, 1645, 1603, 1438, 1362, 1219, 1165, 1144, 1090, 1060, 1036, 977, 940, 763, 705 cm^{-1}; HRMS (ESI): calcd. for $C_{26}H_{34}N_5O_6$ $[M+H]^+$: 512.2504, found 512.2507.

2-Azido-1-[15-(chloroacetyl)-7,11,18,21-tetraoxa-3,15-diazatrispiro[5.2.2.5^{12}.2^{9}.2^{6}]heneicosan-3-yl]ethanone (31c)

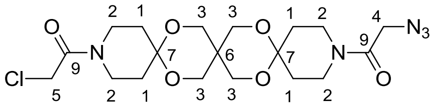

1.17 g (6.66 mmol) 1-[3,3-Bis(hydroxymethyl)-1,5-dioxa-9-azaspiro[5.5]undec-9-yl]-2-chloro-ethanone **27c** [3] were suspended in 200 mL dry Et_2O and cooled to 0°C. 266 mg (6.66 mmol) NaH (60% content) and 850 µL (6.66 µmol) TMSCl were added and the mixture was stirred foe 1h. After this, 2.00 g (6.66 mmol) of diol **29** and a drop TMSOTf were added and stirring was continued over night. On the next day, the mixture was evaporated and the residue was purified by FC (DCM/MeOH 100:2) to give 1.43 g (3.12 mmol, 47%) **31c** as white solid**.** R*f* (DCM/MeOH 100:3) = 0.18; ^{1}H-NMR (300 MHz, $CDCl_3$, ppm): δ 1.83 (m, 8H, H-1), 3.35 (m, 2H, H-2), 3.47 (m, 2H, H-2), 3.58 (m, 4H, H-2), 3.69 (m, 4H, H-3), 3.76 (m, 4H, H-3), 3.91 (s, 2H, H-4), 4.04 (s, 2H, H-5); ^{13}C-NMR (75.5 MHz, $CDCl_3$, ppm): δ 31.0 (C-1), 31.1 (C-1), 33.0 (C-6), 33.2 (C-1), 33.4 (C-1), 38.8 (C-2), 38.9 (C-2), 40.9 (C-5), 41.7 (C-2), 42.9 (C-2), 50.6 (C-4), 63.3 (C-3), 96.6 (C-7), 164.8 (C-9), 165.3 (C-9); m.p. 160 °C; IR: 2966, 2871, 2106, 1649, 1443, 1363, 1266, 1223, 1198, 1165, 1147, 1091, 1036, 940, 887, 793 729 cm^{-1}; HRMS (ESI): calcd. for $C_{19}H_{29}ClN_5O_6$ $[M+H]^+$: 458.1801, found 458.1798.

(1-{2-Oxo-2-[15-(pyren-1-ylacetyl)-7,11,18,21-tetraoxa-3,15-diazatrispiro[5.2.2.5^{12}.2^{9}.2^{6}]heneicos-3-yl]ethyl}-1*H*-1,2,3-triazol-4-yl)methyl-15-(pyren-1-ylacetyl)-7,11,18,21-tetraoxa-3,15-diazatrispiro[5.2.2.5^{12}.2^{9}.2^{6}]heneicosan-3-carboxylate (32a)

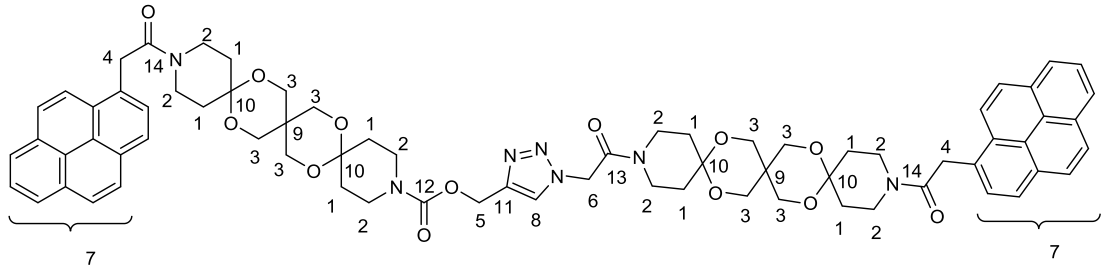

150 mg (240.90 µmol) of azide **31a** and 150 mg (240.90 µmol) of alkyne **30a** were dissolved in 682 µL DCM. 33 µL (240.90 µmol) Et_3N and 12 mg Cu/C catalyst (1.01 mmol/g) were added and the mixture was stirred over night. The mixture was filtered over Celite®, the filter cake was washed with DCM and the combined organic phases were successively washed with 0.02 M HCl and sat. $NaHCO_3$. After drying with $MgSO_4$ and evaporating the residue was purified by FC (DCM/MeOH 100:3) to give (115.53 µmol, 48%) **32a** as white solid. R*f* (DCM/MeOH 100:5) = 0.27; ^{1}H-NMR (300 MHz, $CDCl_3$, ppm): δ 1.48 (m, 4H, H-1), 1.72 (m, 12H, H-1), 3.58 (m, 32H, H-2, H-3), 4.32 (s, 4H, H-4), 5.03 (s, 2H, H-5), 5.21 (s, 2H, H-6), 7.71 (m, 1H, H-7), 7.82 (m, 2H, H-7, H-8), 7.99 (m, 6H, H-7), 8.12 (m, 10H, H-7); ^{13}C-NMR (75.5 MHz, $CDCl_3$, ppm): δ 31.0 (C-1), 31.3 (C-1), 31.5 (C-1), 32.0 (C-1), 32.6 (C-9), 38.4 (C-4), 38.8 (C-2), 40.4 (C-2), 42.5 (C-2), 50.5 (C-5), 58.2 (C-6), 63.0 (C-3), 96.1 (C-10), 96.6 (C-10), 122.6 (C_{ar}H), 124.4 (C_{ar}), 124.7 (C_{ar}H), 124.7 (C_{ar}), 124.9 (C_{ar}H), 125.0 (C_{ar}H), 125.3 (C-8) 125.8 (C_{ar}H), 126.6 (C_{ar}H), 126.9 (C_{ar}H), 127.2 (C_{ar}H), 127.7 (C_{ar}H), 128.8 (C_{ar}), 128.9 (C_{ar}), 130.1 (C_{ar}), 130.5 (C_{ar}), 131.0 (C_{ar}), 143.3 (C-11), 154.6 (C-12), 162.8 (C-13), 169.2 (C-14); m.p. 199.8-210 °C; IR: 3041, 2962, 2868, 1696, 1644, 1467, 1439, 1362, 1233, 1198, 1165, 1143, 1093, 1037, 940, 890, 847, 797, 730, 711, 650 cm^{-1}; HRMS (ESI): calcd. for $C_{72}H_{76}N_7O_{13}$ $[M+H]^+$: 1246,5496, found 1246,5496; ε (MeOH, 341,4 nm) = 89125.094 L/(mol*cm).

[1-(2-Oxo-2-{15-[(2*E*)-3-phenylprop-2-enoyl]-7,11,18,21-tetraoxa-3,15,diazatrispiro[[5.2.2.5^{12}.2^{9}.2^{6}]heneicos-3-yl}ethyl)-1*H*-1,2,3-triazol-4-yl]methyl-15-[(2*E*)-3-phenylprop-2-enoyl]-7,11,18,21-tetraoxa-3,15-diazatrispiro[5.2.2.5^{12}.2^{9}.2^{6}]heneicosan-3-carboxylate (32b)

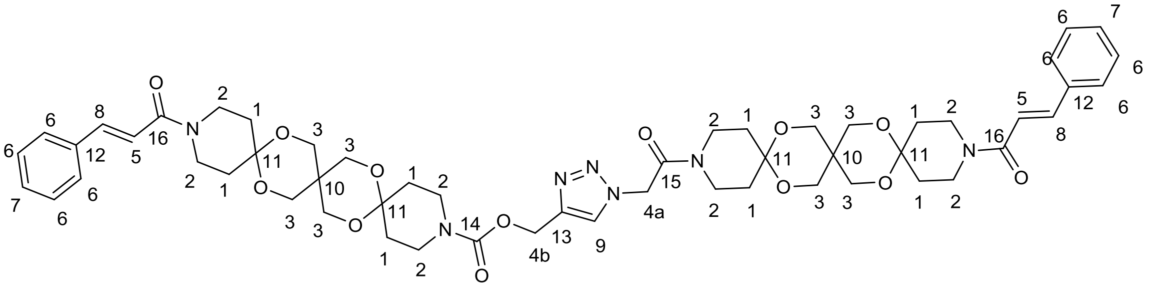

200 mg (391.70 µmol) of azide **31b a**nd 200 mg (391,70 µmol) of alkyne **30b** were dissolved in 1.28 mL DCM. After addition of 54 µL (391.70 µmol) Et_3N and 19 mg Cu/C catalyst (1.01 mmol/g) the mixture was stirred over night. On the next day, the mixture was filtered over Celite®, the filter cake was washed with DCM and the combined organic phases were successively washed with 5 mL 0.02 M HCl and 5 mL sat. $NaHCO_3$. After drying with $MgSO_4$ and evaporating the residue was purified by FC (DCM/MeOH 100:3) to give (258.32 µmol, 66%) **32b** as white solid. R*f* (DCM/MeOH 100:5) = 0.26; ^{1}H-NMR (300 MHz, $CDCl_3$, ppm): δ 1.82 (m, 16H, H-1), 3.42 (m, 5H, H-2), 3.71 (m, 27H, H-2, H-3), 5.19 (s, 4H, H-4), 6.88 (d, ^3J = 15.3 Hz, 2H, H-5), 7.30 (m, 6H, H-6, H-7), 7.45 (m, 4H, H-6), 7.62 (d, ^3J = 15.3 Hz, 2H, H-8), 7.76 (s, 1H, H-9); ^{13}C-NMR (75.5 MHz, $CDCl_3$, ppm): δ 31.1 (C-1), 31.7 (C-1), 32.1 (C-1), 32.9 (C-10), 33.6 (C-1), 39.0 (C-2), 40.5 (C-2), 41.9 (C-2), 42.3 (C-2), 50.7 (C-4a), 58.3 (C-4b), 63.2 (C-3), 96.3 (C-11), 96.8 (C-11), 117.1 (C-5), 125.3 (C-9), 127.5 (C-6), 128.6 (C-6), 129.4 (C-7), 135.1 (C-12), 142.5 (C-8), 143.5 (C-13), 154.7 (C-14), 162.9 (C-15), 165.2 (C-16); m.p. 166.8-172 °C; IR: 2962, 2868, 1696, 1645, 1601, 1468, 1438, 1363, 1230, 1199, 1165, 1143, 1090, 976, 939, 895, 763, 706 cm^{-1}; HRMS (ESI): calcd. for $C_{54}H_{68}N_7O_{13}$ $[M+H]^+$: 1022.4870, found 1022.4864; ε (ACN, 274.2 nm) = 50118.723 L/(mol*cm).

(1-{2-[15-(Chloroacetyl)-7,11,18,21-tetraoxa-3,15-diazatrispiro[5.2.2.5^{12}.2^{9}.2^{6}]heneicos-3-yl]-2-oxoethyl}-1*H*-1,2,3-triazol-4-yl}methyl-15-(chloroacetyl)-7,11,18,21-tetraoxa-3,15-diazatrispiro[5.2.2.5^{12}.2^{9}.2^{6}]heneicosan-3-carboxylate (32c)

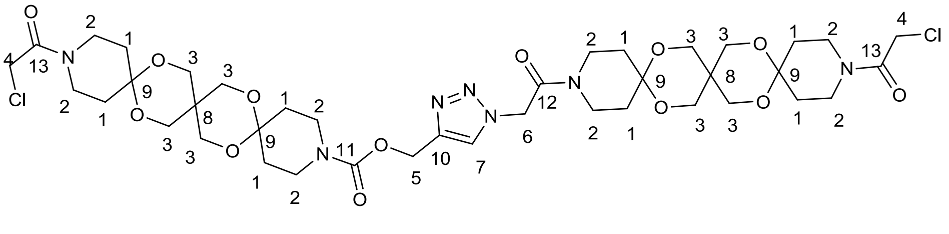

1.3 g (2.85 mmol) of azide **31c** und 1.3 g (2.85 mmol) of alkyne **30c** were dissolved in 5.7 mL DCM. After addition of 592 µL (2.85 mmol) Et_3N and 140 mg Cu/C catalyst (1.01 mmol/g) the mixture was stirred over night. On the next day, the mixture was evaporated and the residue was purified by FC (DCM/MeOH 100:5) to give 1.06 g (1.16 mmol, 41%) **32c** as white solid. R*f* (DCM/MeOH 100:6) = 0.24; ^{1}H-NMR (300 MHz, $CDCl_3$, ppm): δ 1.82 (m, 16H, H-1), 3.44 (m, 10H, H-2), 3.57 (m, 6H, H-2), 3.70 (m, 16H, H-3), 4.03 (s, 2H, H-4), 5.17 (s, 2H, H-5), 5.20 (s, 2H, H-6), 7.75 (s, 1H, H-7); ^{13}C-NMR (75.5 MHz, $CDCl_3$, ppm): δ 31.1 (C-1), 33.0 (C-8), 33.2 (C-1), 38.9 (C-2), 39.1 (C-2), 40.5 (C-2), 40.9 (C-4), 42.0 (C-2), 42.9 (C-2), 50.8 (C-6), 58.3 (C-5), 63.3 (C-3), 96.6 (C-9), 96.8 (C-9), 125.4 (C-7), 143.5 (C-10), 154.7 (C-11), 163.0 (C-12), 164.8 (C-13); m.p. 126-129 °C; IR: 2965, 2870, 1694, 1649, 1469, 1442, 1363, 1231, 1198, 1165, 1145, 1091, 1035, 939, 888, 794, 730, 652 cm^{-1}; HRMS (ESI): calcd. for $C_{40}H_{58}Cl_2N_7O_{13}$ [M+H]$^+$: 914.3464, found 914.3390.

(1-{2-Oxo-2-[15-({[(2*E*)-3-phenylprop-2-enoyl]oxy}acetyl)-7,11,18,21-tetraoxa-3,15-diazatrispiro[5.2.2.5^{12}.2^9.2^6]heneicos-3-yl]ethyl}-1*H*-1,2,3-triazol-4-yl}methyl-15-({[(2*E*)-3-phenylprop-2-enoyl]oxy}acetyl)-7,11,18,21-tetraoxa-3,15-diazatrispiro[5.2.2.5^{12}.2^9.2^6]heneicosan-3-carboxylate (33a)

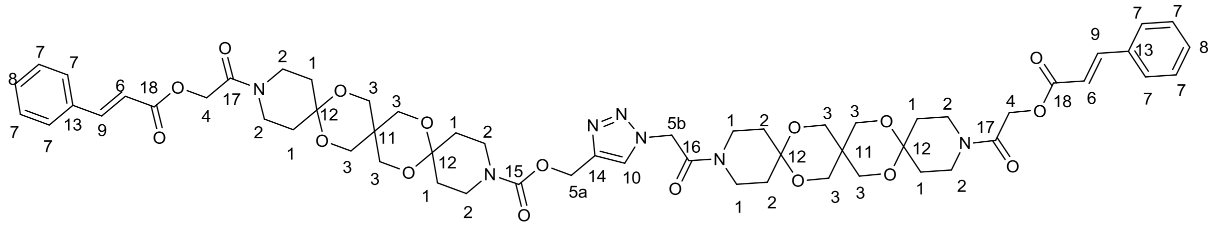

13 mg (191 µmol) KOH and 28 mg (191 µmol) cinnamic acid were dissolved in 3 mL water. The solution was stirred over night, evaporated and the solid was dried in HV. The solid was dissolved in 3 mL dry ACN using an ultrasonic bath. 70 mg (76.50 µmol) **32c** were added under a N_2 atmosphere and the mixture was stirred at 90°C (bath temperature) over night. After evaporation the residue was purified by FC (DCM/MeOH 100:5) to give 48 mg (42.52 µmol, 56%) **33a** as a white solid. R*f* (DCM/MeOH 100:6) = 0.34; ^{1}H-NMR (300 MHz, $CDCl_3$, ppm): δ 1.87 (m, 16H, H-1), 3.45 (m, 8H, H-2), 3.54 (m, 2H, H-2), 3.75 (m, 22H, H-2, H-3), 4.86 (s, 4H, H-4), 5.23 (s, 4H, H-5), 6.57 (d, ^3J = 15 Hz, 2H, H-6), 7.40 (m, 6H, H-7, H-8), 7.53 (m, 4H, H-7), 7.75 (d, ^3J = 15.6 Hz, 3H, H-9, H-10); ^{13}C-NMR (75.5 MHz, $CDCl_3$, ppm): δ 31.0 (C-1), 31.2 (C-1), 33.1 (C-11), 33.1 (C-11), 33.3 (C-1), 33.5 (C-1), 38.8 (C-2), 39.2 (C-2), 40.7 (C-2), 41.3 (C-2), 42.2 (C-2), 51.0 (C-5b), 58.4 (C-5a), 61.5 (C-4), 63.4 (C-3), 96.5 (C-12), 96.9 (C-12), 117.0 (C-6), 125.6 (C-10), 128.2 (C-7), 128.9 (C-7), 130.5 (C-8), 134.2 (C-13), 143.7 (C-14), 146.1 (C-9), 154.9 (C-15), 163.0 (C-16), 164.9 (C-17), 166.4 (C-18); m.p. 133 °C; IR: 2963, 2870, 1704,

1664, 1576, 1446, 1363, 1229, 1312, 1230, 1199, 1166, 1093, 940, 888, 767, 729, 710 cm^{-1}; HRMS (ESI): calcd. for $C_{58}H_{72}N_7O_{17}$ $[M+H]^+$: 1138.4979, found 1138.4961; ε (ACN, 277 nm) = 57543.994 L/(mol*cm).

{1-[2-(15-{[(Anthracen-9-ylacetyl)oxy]acetyl}-7,11,18,21-tetraoxa-3,15-diazatrispiro-[5.2.2.5^{12}.2^{9}.2^{6}]heneicos-3-yl]-2-oxoethyl}-1*H*-1,2,3-triazol-4-yl}methyl-15-{[(anthracen-9-yl-acetyl)oxy]acetyl}-7,11,18,21-tetraoxa-3,15-diazatrispiro[5.2.2.5^{12}.2^{9}.2^{6}]heneicosan-3-carboxylate (33b)

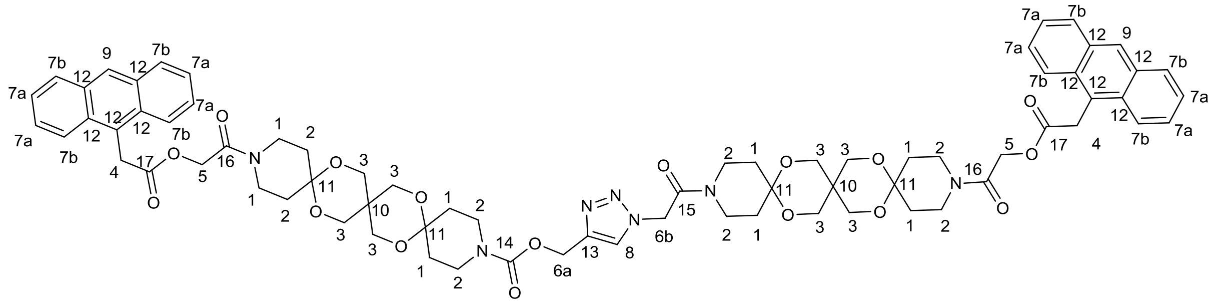

10 mg (150 µmol) KOH and 36 mg (150 µmol) 9-anthraceneacetic acid were dissolved in 3 mL water. The solution was stirred over night, evaporated and the solid was dried in HV. The solid was dissolved in 3 mL dry ACN by means of a ultrasonic bath. 55 mg (60 µmol) **32c** were added under a N_2 atmosphere and the mixture was stirred at 90°C (bath temperature) over night. After evaporation the residue was purified by FC (DCM/MeOH 100:5) to give 31 mg (22.75 µmol, 39%) **33b** as a white solid. R*f* (DCM/MeOH 100:8) = 0.41; ^{1}H-NMR (300 MHz, $CDCl_3$, ppm): δ 1.61 (m, 2H, H-1), 1.78 (m, 14H, H-1), 3.17 (m, 4H, H-2), 3.51 (m, 9H, H-2), 3.69 (m, 19H, H-2, H-3), 4.70 (s, 4H, H-4), 4.78 (s, 4H, H-5), 5.20 (s, 4H, H-6a, H-6b), 7.47 (m, 4H, H-7a), 7.56 (m, 4H, H-7a), 7.79 (s, 1H, H-8), 8.02 (d, ^3J = 5,1 Hz, 4H, H-7b), 8.32 (d, ^3J = 5.4 Hz, 4H, H-7b), 8.44 (s, 2H, H-9); ^{13}C-NMR (75.5 MHz, $CDCl_3$, ppm): δ 31.1 (C-1), 31.2 (C-1), 32.9 (C-1), 33.0 (C-10), 33.1 (C-10), 33.3 (C-1), 33.7 (C-5), 38.8 (C-2), 39.2 (C-2), 40.7 (C-2), 41.1 (C-2), 42.1 (C-2), 51.0 (C-6b), 58.4 (C-6a), 61.9 (C-4), 63.3 (C-3), 96.6 (C-11), 96.7 (C-11), 124.3 (C-7b), 125.0 (C-7a), 125.5 (C-8), 126.3 (C-7a), 127.5 (C-9), 128.2 (C-12), 128.9 (C-12), 129.1 (C-7b), 130.6 (C-12), 131.5 (C-12), 144.8 (C-13), 154.9 (C-14), 163.0 (C-15), 164.6 (C-16), 171.0 (C-17); m.p. 148-153 °C; IR: 2933, 2868, 1740, 1694, 1663, 1443, 1362, 1230, 1198, 1163, 1092, 939, 887, 791, 732 cm^{-1}; HRMS (ESI): calcd. for $C_{72}H_{79}N_7NaO_{17}$ $[M+Na]^+$: 1336.5425, found 1336.5542; ε (ACN, 393.4 nm) = 10232.9 L/(mol*cm).

{1-[2-Oxo-2-(15-{[(pyren-1-ylacetyl)oxy]acetyl}-7,11,18,21-tetraoxa-3,15-diazatrispiro-[5.2.2.5^{12}.2^{9}.2^{6}]heneicos-3-yl)ethyl]-1*H*-1,2,3-triazol-4-1}methyl-15-{[(pyren-1-ylacetyl)oxy]acetyl}-7,11,18,21-tetraoxa-3,15-diazatrispiro[5.2.2.5^{12}.2^{9}.2^{6}]heneicosan-3-carboxylate (33c)

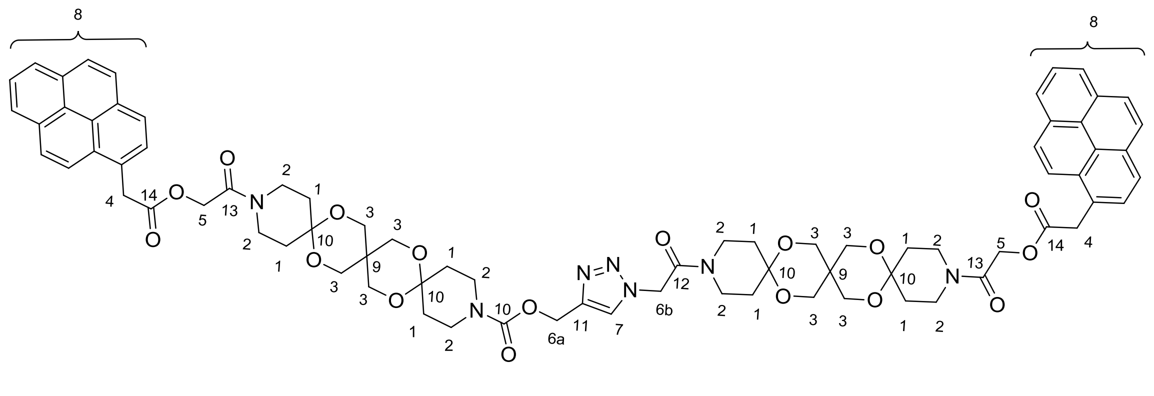

12 mg (180 µmol) KOH and 47 mg (180 µmol) 1-pyrenylacetic acid were dissolved in 3 mL water. The solution was stirred over night, evaporated and the solid was dried in HV. The solid was dissolved in 3 mL dry ACN using an ultrasonic bath. 55 mg (60 µmol) **32c** were added under a N_2 atmosphere and the mixture was stirred at 90°C (bath temperature) over night. After evaporation the residue was purified by FC (DCM/MeOH 100:5) to give 64 mg (47 µmol, 78%) **33c** as a white solid. R*f* (DCM/MeOH 100/8) = 0.43; ^{1}H-NMR (300 MHz, $CDCl_3$, ppm): δ 1.44 (m, 4H, H-1), 1.72 (m, 12H, H-1), 3.06 (m, 4H, H-2), 3.59 (m, 28H, H-2, H-3)), 4.45 (s, 4H, H-4), 4.69 (s, 4H, H-5), 5.12 (s, 2H, H-6a), 5.22 (s, 2H, H-6b), 7.76 (s, 1H, H-7), 7.96-8.27 (m, 20H, H-8); ^{13}C-NMR (75.5 MHz, $CDCl_3$, ppm): δ 31.2 (C-1), 31.2 (C-1), 31.5 (C-1), 32.4 (C-9), 38.7 (C-2), 39.0 (C-4), 39.1 (C-2), 40.6 (C-2), 42.0 (C-2), 50.8 (C-6b), 58.4 (C-6a), 62.1 (C-5), 63.1 (C-3), 96.5 (C-10), 96.8 (C-10), 123.2-125.2 (C-Ar), 125.4 (C-7), 125.9-131.2 (C-Ar), 143.6 (C-11), 154.8 (C-10), 163.0 (C-12), 164.5 (C-13), 171.0 (C-14); m.p. 170 °C; IR: 2927, 2856, 1739, 1693, 1662, 1440, 1362, 1339, 1229, 1163, 1146, 1092, 1047, 939, 889, 845, 729 cm^{-1}; HRMS (ESI): calcd. for $C_{76}H_{79}N_7NaO_{17}$ [M+Na]$^+$: 1384.5425, found 1384.5417.

(1-{2-[15-(Azidoacetyl)-7,11,18,21-tetraoxa-3,15-diazatrispiro[5.2.2.5^{12}.2^{9}.2^{6}]heneicos-3-yl]-2-oxoethyl}-1*H*-1,2,3-triazol-4-yl}methyl-15-(azidoacetyl)-7,11,18,21-tetraoxa-3,15-diazatrispiro[5.2.2.5^{12}.2^{9}.2^{6}]heneicosan-3-carboxylate (34)

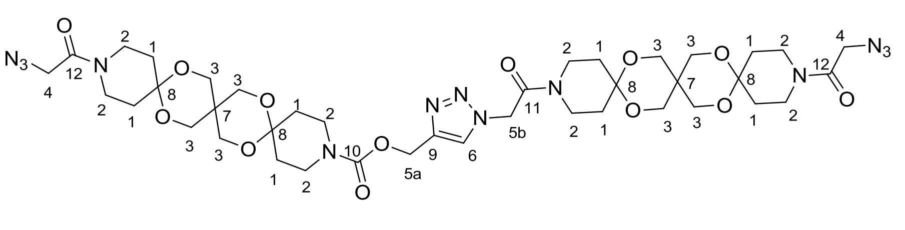

50 mg (54.66 µmol) **32c** and 9 mg NaN_3 were dissolved in 200 µL dry DMF and stirred over night. After evaporation and purification by FC (DCM/MeOH 100:6) of 44 mg (47.41 µmol, 87%) **34** were obtained as white solid. R*f* (DCM/MeOH 100:6) = 0.20; ^{1}H-NMR (300 MHz, CD $CDCl_3$, ppm): δ 1.85 (m, 16H, H-1), 3.36 (m, 4H, H-2), 3.46 (m, 4H, H-2), 3.54 (m, 2H, H-2), 3.62 (m, 6H, H-2), 3.73 (m, 16H, H-3), 3.93 (s, 2H, H-4), 5.21 (m, 4H, H-5a, H-5b), 7.79 (s, 1H, H-6); ^{13}C-NMR (75.5 MHz, $CDCl_3$, ppm): δ 31.0 (C-1), 31.2 (C-1), 33.1 (C-7), 33.2 (C-1), 33.5 (C-1), 38.9 (C-2), 39.2 (C-2), 40.6 (C-2), 41.8 (C-2), 42.1 (C-2), 50.7 (C-4), 50.9 (C-5b), 58.4 (C-5a), 63.4 (C-3), 96.7 (C-8), 96.9 (C-8), 125.4 (C-6), 143.6 (C-9), 154.8 (C-10), 163.0 (C-11), 165.4 (C-12); m.p. 121.9-123°C; IR: 2962, 2871, 2105, 1693, 1653, 1443, 1364, 1340, 1228, 1198, 1165, 1144, 1092, 1037, 940, 909, 886, 796, 729 cm^{-1}; HRMS (ESI): calcd. for $C_{40}H_{58}N_{13}O_{13}$ $[M+H]^+$: 928.4272, found 928.4270.

[1-(2-{15-[(4-{[9-(2,5-Di-tert-butylphenyl)-2,8,9,10-tetrahydroisochino [4',5',6':6,5,10]anthra[2,1,9-def]isochinolin-2(1*H*)-yl]methyl)-1*H*-1,2,3-triazol-1-yl)acetyl]-7,11,18,21-tetraoxa-3,15-diazatrispiro[5.2.2.5^{12}.2^9.2^6]heneicos-3-yl}-2-oxoethyl)-1*H*-1,2,3-triazol-4-yl)methyl-15-[(4-{[9-(2,5-di-tert-butylphenyl)-3,8,9,10-{N-[(2*E*)-3-phenylprop-2-enoyl]glycyl}-7,11,18,21-tetraoxa-3,15-tetrahydroisochino [4',5',6':6,5,10]anthra[2,1,9-*def*]isochinolin-2(1*H*)-yl]methyl)-1*H*-1,2,3-triazol-1-yl)acetyl]-7,11,18,21-tetraoxa-3,15-diazatrispiro[5.2.2.5^{12}.2^9.2^6]heneicosan-3-carboxylate (36)

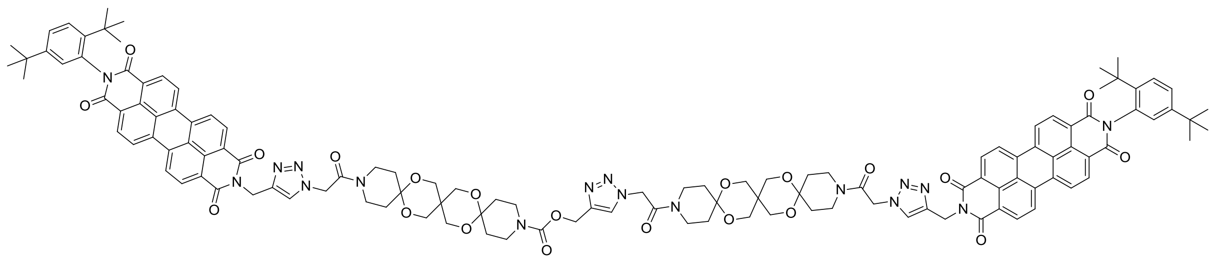

11 mg (12 µmol) **35** [4] and 15 mg (24 µmol) **34** were dissolved in 96 µL DCM. 4.5 µL (32 µmol) Et_3N and a spatula tip-full of Cu/C catalyst (1.01 mmol/g) were added. After stirring over night the catalyst was removed by filtration, the filtrate was evaporated and the residue was purified by FC (DCM/MeOH 100:5) to give 16 mg (7.5 µmol, 62%) **36** as red solid. MS (Maldi-TOF, matrix: α-cyano-4-hydroxycinnamic acid): calcd. for $C_{122}H_{121}N_{17}NaO_{21}$ $[M+Na]^+$: 2185, found: 2187; VIS-absorption λ_{MAX} = 527 nm, emission λ_{MAX} = 538 nm.

2-(Diphenylphosphan)phenyl (2*E*)-3-phenylprop-2-enoate (38)

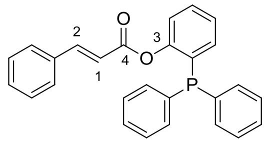

500 mg (1.80 mmol) 2-(Diphenylphosphanyl)phenol were dissolved in 12 mL dry pyridine. 359 mg (2,16 mmol) cinnamoyl chloride and 22 mg (180 µmol) DMAP were added. The mixture was stirred for 2h at r.t. and then evaporated. After removing traces of solvent in HV 50mL DCM were added and the solution was successively washed with 40 mL HCl (10% content) and sat. $NaHCO_3$. After drying with $MgSO_4$ and evaporation the residue was purified by FC (DCM/MeOH 100:2) to give (1.18 mmol, 65%) **38** as white solid. R*f* (DCM/MeOH 100/4) = 0.65; ^{1}H-NMR (300 MHz, $CDCl_3$, ppm): δ 6.40 (d, ^3J = 16.2 Hz, 2H, H-1), 6.89 (m, 1H, H_{-Ar}), 7.36 (m, 19H, H-Ar); ^{13}C-NMR (75.5 MHz, $CDCl_3$, ppm): δ 116.9 (C-1), 122.5-135.7 (CH-Ar, C-Ar), 146.2 (C-2), 152.9 (C-3), 164.4 (C-4); ^{31}P-NMR (75.5 MHz, $CDCl_3$, ppm): δ -14.2.; m.p. 143.5-145 °C; IR: 3055, 1728, 1631, 1431, 1306, 1190, 1127, 1063, 965, 749, 697 cm^{-1}; HRMS (EI): calcd. for $C_{27}H_{21}O_2P_1$ $[M+H]^+$: 408.1279, found 408.1298.

{1-[2-Oxo-2-(15-{*N*-[(2*E*)-3-phenylprop-2-enoyl]glycyl}-7,11,18,21-tetraoxa-3,15-diazatrispiro[5.2.2.5^{12}.2^9.2^6]heneicos-3-yl)ethyl]-1*H*-1,2,3-triazol-4-yl}methyl-15-{*N*-[(2*E*)-3-phenylprop-2-enoyl]glycyl}-7,11,18,21-tetraoxa-3,15-diazatrispiro[5.2.2.5^{12}.2^9.2^6]heneicosan-3-carboxylate (39)

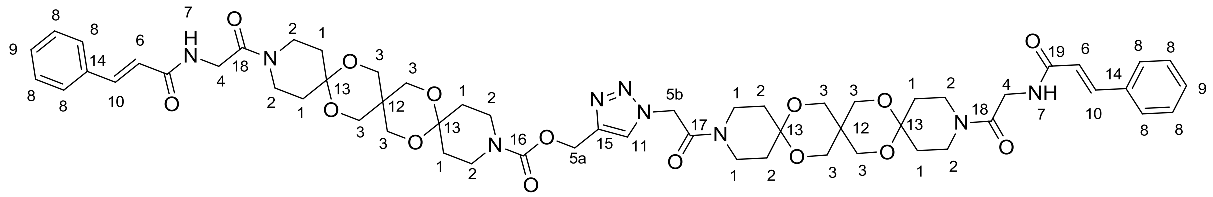

20 mg (21.5 µmol) **34** and 22 mg (53.9 µmol) **38** were dissolved in 1 ml DMF. The mixture was refluxed (bath temp. 120°C) and then quenched with water. After evaporation the mixture was purified by FC (DCM/MeOH 100:5) to give 6 mg (5.3 µmol, 25%) **39** as white solid. R*f* (DCM/MeOH 100:8) = 0.38; ^{1}H-NMR (300 MHz, $CDCl_3$, ppm): δ 1.86 (m, 16H, H-1), 3.45 (m, 6H, H-2), 3.60 (m, 5H, H-2), 3.67 (m, 5H, H-2, H-3), 3.76 (m, 16H, H-3), 4.19 (s, 4H, H-4), 5.23 (m, 4H, H-5a, H-5b), 6.50 (d, ^3J = 9.3 Hz, 2H, H-6), 6.81 (s, 2H, H-7), 7.37 (m, 6H, H-8, H-9), 7.52 (m, 4H, H-8), 7.61 (d, ^3J = 9.3 Hz, 2H, H-10), 7.80 (s, 1H, H-11); ^{13}C-NMR (75.5 MHz, $CDCl_3$, ppm): δ 31.7 (C-1), 32.9 (C-1), 33.2 (C-12), 34.4

(C-1), 38.9 (C-2), 40.7 (C-2), 41.2 (C-2), 41.5 (C-4), 51.1 (C-5b), 58.4 (C-5a), 63.4 (C-3), 96.7 (C-13), 96.7 (C-13), 120.2 (C-6), 125.5 (C-11), 127.8 (C-8), 128.8 (C-8), 129.8 (C-9), 134.7 (C-14), 141.2 (C-10), 146.6 (C-15), 154.9 (C-16), 163.06 (C-17), 165.7 (C-18), 166.2 (C-19); IR: 2926, 1654, 1443, 1341, 1222, 1090, 939, 891, 803, 748, 663 cm^{-1}; HRMS (ESI): calcd. for $C_{58}H_{74}N_9O_{15}$ $[M+H]^+$: 1136.5299, found 1136.5327; ε (ACN, 271 nm) = 27542.3 L/(mol*cm).

***N*-(Anthracen-9-ylacetyl)-L-histidine methylester (41)**

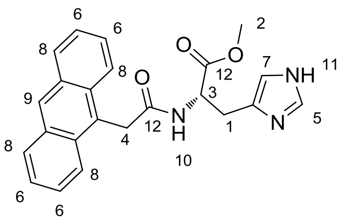

400 mg (1.69 mmol) 9-anthraceneacetic acid were dissolved in 5 mL DMA. 410 mg (1.69 mmol) L-histidine-methylester dihydrochloride, 1,06 g (2.03 mmol) PYBOP and 1,15 mL (6.77 mmol) DIEA were added and the mixture was stirred over night. The solution was quenched with 6 mL water and extracted twice with DCM (20 mL, 10 mL). The combined extracts were dried with $MgSO_4$, evaporated in HV and the residue was purified by FC (DCM/MeOH 100:6) to give (0.76 mmol, 45%) **41** as solid. R*f* (DCM/MeOH 100:8) = 0.15; ^{1}H-NMR (300 MHz, $CDCl_3$, ppm): δ 2.96 (m, 2H, H-1), 3.57 (s, 3H, H-2), 4.50 (m, 1H, H-3), 4.55 (s, 2H, H-4), 6.88 (s, 1H, H-5), 7.53 (m, 5H, H-5, H-6), 8.06 (d, ^3J = 9.6 Hz, 2H, H-8), 8.24 (d, ^3J = 9.3 Hz, 2H, H-8), 8.53 (s, 1H, H-9), 8.77 (d, ^3J = 7.5 Hz, 1H, H-10); ^{13}C-NMR (75.5 MHz, $CDCl_3$, ppm): δ 28.9 (C-1), 34.0 (C-4), 51.7 (C-2), 52.5 (C-3), 116.5 (C-5), 124.9 (CH-Ar), 125.6 (CH-Ar), 126.1 (C-9), 128.4 (C-Ar), 128.6 (CH-Ar), 130.27 (C-Ar), 130.9 (C-Ar), 133.1 (C-Ar), 134.8 (CH-Ar), 170.0 (C-12), 172.0 (C-12); m.p. 198-206,5 °C; $[\alpha]_D^{23}$ = -0.6 (c=0.44, DMSO); IR: 3185, 3010, 2888, 1729, 1647, 1590, 1562, 1523, 1488, 1442, 1362, 1296, 1255, 1230, 1175, 1155, 1085, 1005, 980, 947, 841, 731, 620 cm^{-1}; HRMS (ESI): calcd. for $C_{23}H_{22}N_3O_3$ $[M+H]^+$: 388.1656, found 388.1656.

(1-{2-[15-({[(2*S*)-2-[(Anthracen-9-ylacetyl)amino]-3-(1*H*-imidazol-4-yl)propanoyl]oxy}acetyl)-7,11,18,21-tetraoxa-3,15-diazatrispiro [5.2.2.5^{12}.2^{9}.2^{6}]heneicos-3-yl]-2-oxoethyl}-1*H*-1,2,3-triazol-4-yl)methyl-15-({[(2*S*)-2-[(anthracen-9-ylacetyl)amino]-3-(1*H*-imidazol-4-yl)propanoyl]oxy)acetyl)-7,11,18,21-tetraoxa-3,15-diazatrispiro[5.2.2.5^{12}.2^{9}.2^{6}]heneicosan-3-carboxylate 43

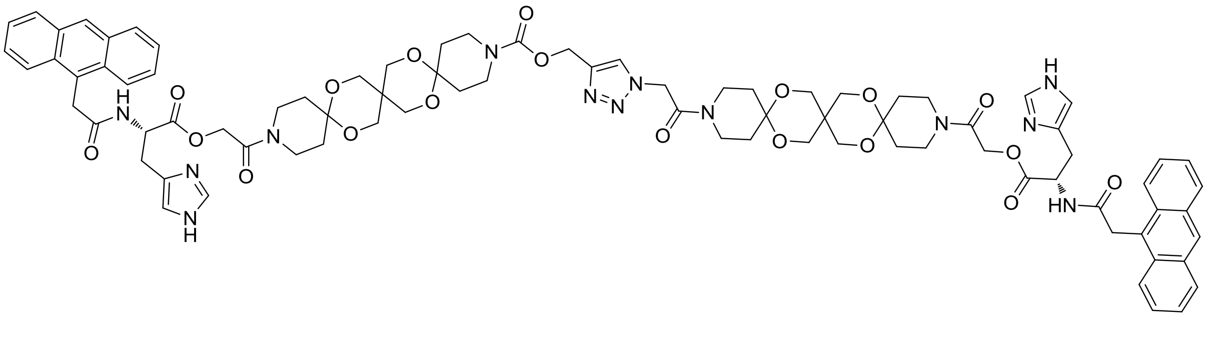

26 mg (394 µmol) KOH and 153 mg (395 µmol) **41** were dissolved in 8 mL water. The solution was refluxed over night, evaporated and dried in HV to give the potassium salt of **42**. The salt was suspended in 8mL dry CAN and treated with an ultrasonic bath. After this, 120 mg (131 µmol) **32c** were added under a N_2 atmosphere and the mixture was refluxed over night (bath temp. 90°C). On the next day the precipitated solid was filtered off and washed with ACN to give 208 mg (131 µmol, 100%) as solid. Due to the scarce solubility only a MALDI-TOF-MS could measured (matrix: matrix α-cyano-4-hydroxycinnamic acid) calcd. for $C_{84}H_{94}N_{13}O_{19}$ $[M+H]^{+}$: 1588.6789, found 1588.6.

Irradiation spectra

Compounds **32b**, **33a**, **39** were irradiated in ACN.

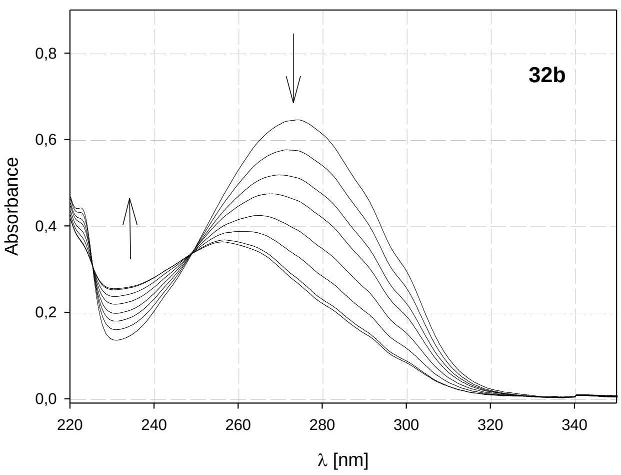

Figure SI-1

c = 0.01413 mmol/L

t = 0; 0.5; 1; 1.5; 2.5; 4; 8; 16 min

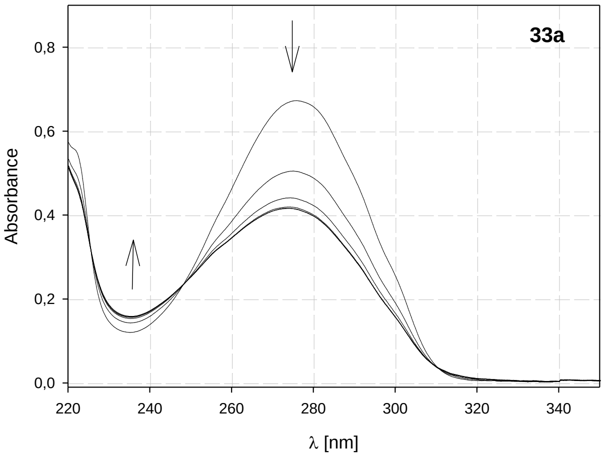

Figure SI-2

c = 0.01413 mmol/L

t = 0; 0.25; 0.5; 1; 1.5; 3 min

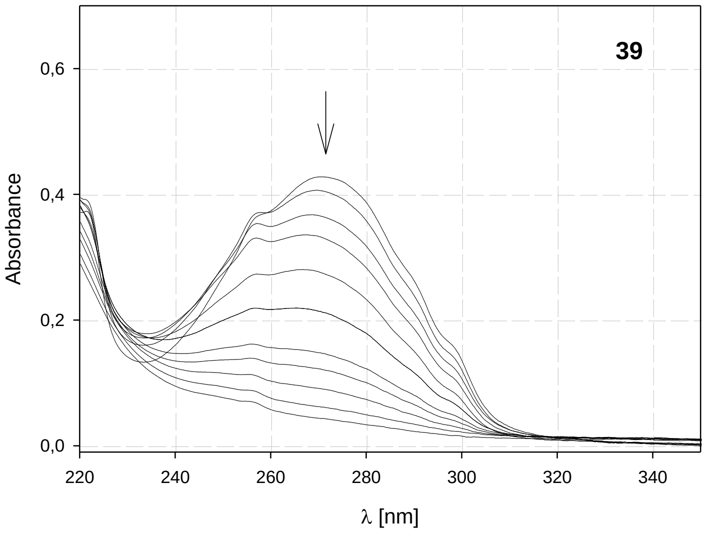

Figure SI-3

c = 0.01413 mmol/L

t = 0; 0.33; 1; 6; 27; 67; 127; 157; 217; 337; 517 min

Spectra of the rod in DOPC MLVs

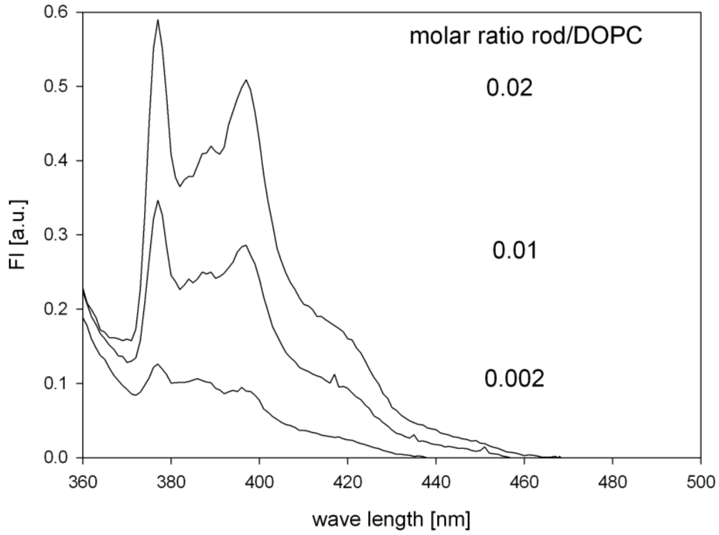

Figure SI-4

Fluorescence spectra of **32a** in DOPC membranes. Multilamellar vesicles (MLVs) were prepared containing DOPC and **32a** at different molar ratios. The fluorescence spectra (λ_{ex} = 340 nm) of MLVs were recorded at 37 °C.

Quenching of fluorescence in DOPC/rod SUVs by KJ

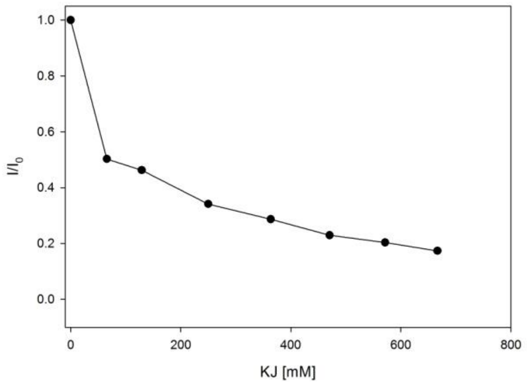

Figure SI-5

Quenching of the fluorescence of **32a** in DOPC vesicles by KJ. Fluorescence spectra of small unilamellar vesicles (SUVs) containing DOPC and 32a were recorded (λex = 340 nm) at 37 °C in the absence and in the presence of various concentrations of KJ. Fluorescence intensities at 377 nm in the presence of KJ were related to that in the absence of KJ.

MD simulation

The MD simulation was performed with the program MOE 2010.10 (Chemical Comuting Group) using the MMFF94x forcefield [5] at 600 K. For using MD simulations to evaluate the rigidity of molecular rods see [6].

End-to-End distance distribution of a model articulated rod (**Figure SI-6**):

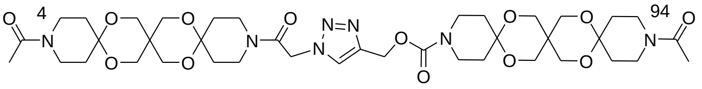

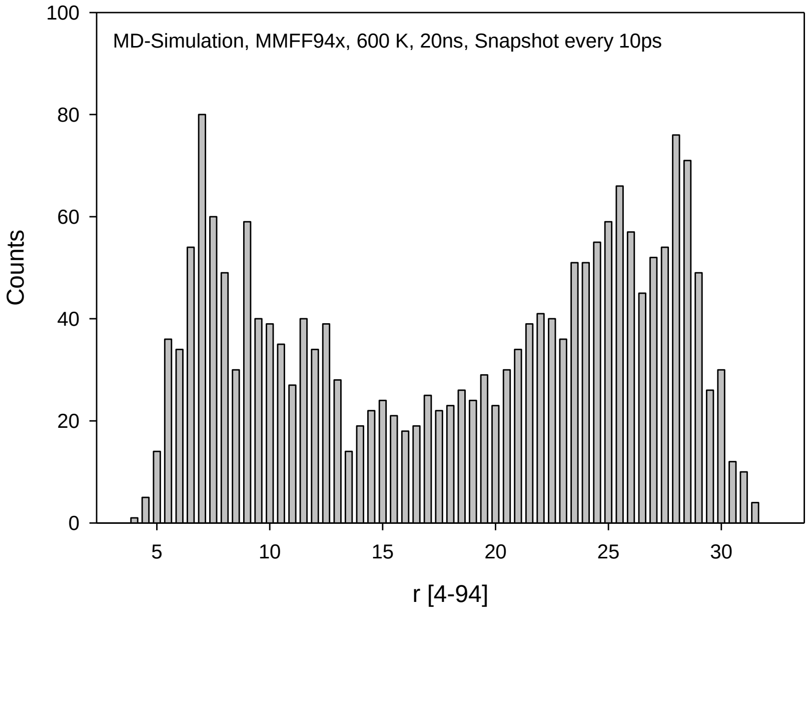

Materials and Methods

Materials

1,2-Dioleoyl-sn-glycero-3-phosphocholine (DOPC) was purchased from Avanti Polar Lipids (Alabaster, AL). HEPES buffered salt solution (HBS) contained 10 mM HEPES and 145 mM NaCl, pH 7.4.

Preparation of multilamellar vesicles (MLVs)

Aliquots of DOPC (from a stock solution in chloroform and methanol) and 32a (from a stock solution in methanol) were transferred to a glass tube and resuspended in HBS to give a final lipid concentration of 0.5 mM and different rod concentrations. The suspensions were vigorously vortexed resulting in the formation of MLVs.

Fluorescence spectroscopy

Fluorescence spectra were recorded at an Aminco Bowman 2 fluorescence spectrophotometer (Thermo electron corporation, Langenselbold, Germany).

References

[1] Lipshutz, B. H.; Taft, B. R. *Angew. Chem.* **2006**, *118*, 8415-8418.

[2] Kobayashi, M.; Kurosu, M.; Wang, W. Q.; Kitagawa, I. *Chem. Pharm. Bull.* **1994**, *42*, 2394-2396.

[3] Wessig, P.; Möllnitz, K. *J. Org. Chem.* **2008**, *73*, 4452-4457.

[4] Techen, A.; Czapla, S.; Möllnitz, K.; Budach, D.; Wessig, P.; Kumke, M.U. *Helv. Chim. Acta* **2013**, *96*, 2046 – 2067.

[5] Halgren, T. A. *J. Comp. Chem.* **1996,** 490-519.

[6] Wessig, P.; Möllnitz, K. *J. Org. Chem.* **2012**, *77*, 3907 – 3920.
